# Sea Buckthorn, Aronia, and Black Currant Pruning Waste Biomass as a Source of Multifunctional Skin-Protecting Cosmetic and Pharmaceutical Cream Ingredients

**DOI:** 10.3390/ijms27020701

**Published:** 2026-01-09

**Authors:** Anna Andersone, Anna Ramata-Stunda, Natalija Zaharova, Liga Petersone, Gints Rieksts, Uldis Spulle, Galina Telysheva, Sarmite Janceva

**Affiliations:** 1 Lignin Chemistry Laboratory, Latvian State Institute of Wood Chemistry, Dzerbenes Street 27, LV-1006 Riga, Latvia; natalija.zaharova@gmail.com (N.Z.); gints.rieksts@inbox.com (G.R.); uldis.spulle@lbtu.lv (U.S.); 2 Faculty of Medicine and Life Sciences, University of Latvia, Jelgavas Street 1, LV-1004 Riga, Latvia; anna.ramata-stunda@lu.lv; 3 Laboratory of Finished Dosage Forms, Riga Stradinš University, Dzirciema 16, LV-1007 Riga, Latvia; liga.petersone@rsu.lv; 4 The Institute of Physics, Faculty of Science and Technology, University of Latvia, Miera Street 32, LV-2169 Salaspils, Latvia; 5 Institute of Civil Engineering and Woodworking, Latvia University of Life Sciences and Technologies, Dobeles Street 41, LV-3001 Jelgava, Latvia

**Keywords:** lignocellulosic agro-waste, sea buckthorn, aronia, blackcurrant, oxidative stability, skin protective cosmetic cream, pharmaceutical topical formulation

## Abstract

Fruit shrubs’ lignocellulosic biomass remaining as waste after harvesting and/or after pruning is an underutilized, little-explored bioresource. Sea buckthorn (*Hippophae rhamnoides* L.), aronia (*Aronia melanocarpa*) and blackcurrant (*Ribes nigrum*) berries are rich in biologically active compounds, so these shrubs’ woody biomass derivatives are prospective investigation objects. The influence of pre-treated biomass, extracts, and purified proanthocyanidins on the oxidative stability of lipid-based systems was studied by accelerated oxidation method. Emulsion stability, antimicrobial activity against bacteria that causes acne—*Cutibacterium acnes*; contaminating wounds; skin care products—*Streptococcus pyogenes*, *Pseudomonas aeruginosa*, *Staphylococcus aureus*, and *Bacillus cereus*; cytotoxicity and phototoxicity of extracts and proanthocyanidins on HaCaT human keratinocytes were tested. The study established that biomass, lipophilic extracts obtained using liquefied hydrofluorocarbon, and hydrophilic extracts obtained by aqueous ethanol increased oxidative stability of lipid-based formulations. Compounds with skin-protecting properties were detected. Sea buckthorn and aronia hydrophilic extracts and proanthocyanidins had the highest antimicrobial activity. Low phototoxicity was revealed, emphasizing safety and applicability in topical formulations; human HaCaT keratinocyte viability was the best with aronia extracts, but none of the other samples decreased cell viability by more than 50%. It was proven that agro-waste biomass is a prospective source of multifunctional ingredients for cosmetic and pharmaceutical topical formulations.

## 1. Introduction

Fruit tree pruning waste comprises twigs, with bark and with or without leaves, depending on the season, cut from the trees annually or during rejuvenation to improve fruit quality, yield, tree health, and shrub tree size. According to different sources, fruit tree pruning waste account for around 2–25 mln tones [1], and at the moment 90% of it in the EU, is burned [2], releasing 1–2 kg of CO_2_ per kg of dry biomass.

Sea buckthorn (*Hippophae rhamnoides*, L., SBT) and aronia, or chokeberry (*Aronia melanocarpa*, AR), the deciduous shrub trees, can grow in extreme climates and difficult soils, both wet and sandy, adapting to each environment. Aronia tolerates both wet and dry soils, survives in sandy dunes, dry rocky slopes, dry bluffs and balds. In 2020 SBT had been reported to grow in 52 countries, occupying globally an area of 2.33 mln ha [3]. Thailand, Canada, Malaysia, and Poland are the 4 top SBT berries exporters in 2023 (export values $760.7 M, $491.6 M and $287.2) [4], followed by Peru, Chile, USA, Serbia, Netherlands and Belgium. Native to eastern North America [5], AR is originally a small shrub; its orchards’ trees grow 1.8 to 2.4 m tall. Now this tree with extremely healthy berry with high polyphenols content is gaining increased attention in Europe, where Poland is the largest producer [6], and USA [7]. The industrial harvesting of SBT berries by cutting the whole branch results in a large amount of agro-waste (20% of berries mass). Both SBT and AR shrub trees demand yearly pruning or removing the plant suckers around the base of the shrub. Plantations of SBT demand the full cut of the whole shrub every 4th year, and AR demands renewal cut every 8–10 years, to rejuvenate an orchard, for easier harvesting and better quality of the berries [8]. The precise statistical data of pruning waste from SBT and AR trees are absent, but if compare the amount of biomass cut from one tree with, e.g., apple trees, SBT and AR pruning waste could reach around 10 kg from one tree per year, 1.5 tons of dry-weight pruning biomass per ha on average.

Another under investigated fruit crop pruning lignocellulosic biomass is the one of blackcurrant (BC), demanding yearly pruning since the biggest amount of the berries grow on young branches [9]. Woody biomass of BC could amount 6000 kg ha^−1^ annually [10]. Rejuvenation—cutting of the entire shrub—pruning has to be performed every 5–10 years and can produce 10–20 tonnes·ha^−1^, or 5–10 tonnes·ha^−1^ of dry weight lignocellulosic biomass. One BC shrub can generate 1–2 kg of pruning waste annually (20–30% of the shrub biomass), and 15–30 kg after rejuvenation.

Finding an application for this pruning biomass is necessary for waste-free SBT, AR, and BC technological processing. This agro-waste could become a stable source of raw material available for the bioeconomy. Berries of SBT, AR, and BC are extremely rich in bioactive compounds, nearly 200 are reported for SBT [11,12]. The amount of anthocyanins and flavonoids in the berry of AR is five times higher than cranberry and blueberry, it also contains strong anticancer compounds [13]. Oil of SBT is used in pharmacology, food, cosmetics, and even for skin protection from ionizing radiation [14,15]. Significant amount of carotenoids and tocopherols (lipophilic antioxidants) as well as polyphenols and ascorbic acid (hydrophilic antioxidants) are present in SBT [16]. Aronia rarely seems to be affected by insects and thus could be both a low-maintenance crop and a source of powerful antimicrobials [13]. Berries of BC have proven anti-microbial activity as well [17]. Antioxidant properties of these plants’ berries are reported, including protection against UV irradiation, revitalization of wounds and skin burns [18,19,20,21,22]. Pulp and oil of SBT are used in cosmetic preparations and for treating various skin disorders [23,24].

In the previous studies, it was found that SBT and AR biomass contain a wide range of bioactive compounds, including polyphenolic compounds, with the antioxidant properties [25,26,27,28]. The research on the lignocellulosic biomass of these shrub trees is very limited. Their application in skin care formulations seems promising since skin care and medical formulations need replacement of the fossil-based ingredients with reported side effects. The skin serves as the primary interface between body and environment and as a barrier against the entry of microbes [29]. Market size of skin care products’ was valued globally as USD 130.50 billion, for topical medicinal formulations—as USD 190.5 billion in 2024 with expected annual growth of 6% from 2025 to 2030 [30]. Lipids, one of the main ingredients in creams, are susceptible to oxidation that declines efficiency and shelf life [31,32]. Volatile lipid oxidation products can affect also product odor [33]. Antioxidants in creams formulations serve two purposes: protecting the cream from oxidative damage and protecting the skin. The most common synthetic antioxidants today are butylated hydroxyanisole and hydroxytoluene, and the “green” ones—tocopherols and ascorbic acid [34]. The European Green Deal concept of avoiding the loss of resources promotes finding natural functional ingredients without harm during production and in application [35].

Another emerging problem in the skin care and topical formulations industry is microbial contamination [36]. It can result both in breaking down active ingredients and a serious health threat to consumers [37], especially with atopic dermatitis, susceptible to recurrent microbial infections [38]. Previous studies have shown that among the pathogens that pose problems for humans, and with which it is possible to deal with the use of cosmetic preparations, the most frequent are *Pseudomonas aeruginosa*, *Staphylococcus aureus*, and *Bacillus* species [39,40,41]. *Streptococcus pyogenes* is one of the main bacterial causes of skin and soft tissue infections worldwide [42]. The oxidation of sebum and *Cutibacterium acnes* bacteria is the main cause of inflammatory acne [43]. Thus, this study reveals the antibacterial activity of SBT, AR, and BC agro-waste biomass derivatives against these bacteria.

Microbial infections, as well as oxidative stress in most cases, is accompanied by inflammation. One of the main inflammatory response mediators is Interleukin-8 (IL-8) [44]. In the authors’ previous research [26], it was shown that SBT agro-waste-derived proanthocyanidins (PACs) reduce the IL-8 protein secretion. Accumulation of IL-8 in the skin in response to inflammatory stimuli causes damage to epidermal stem cells, decreases the expression of bleomycin hydrolase, a moisturizing factor-producing enzyme, and, thus, deteriorates skin barrier function and accelerates cell aging [45,46], thus, adding PACs in cream could have anti-inflammatory and, correspondingly, anti-aging effects. Similarly, AR berries polyphenols have improved arterial function [47].

**Figure 1:**
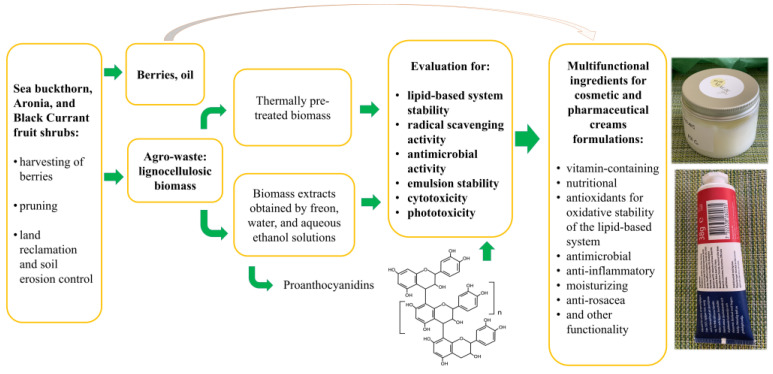
Validation of SBT, AR, and BC biomass as a source of multifunctional ingredients in topical formulations.

Considering the above-mentioned, the aim of this study was characterizing and validating SBT, AR, and BC agro-waste biomass and its’ derived extracts and PACs for multipurpose application in topical formulations for cosmetic and medicinal creams, allowing cascading use of berries and lignocellulosic parts, and obtaining various added-value products for the replacement of various synthetic oil-derived ingredients (Figure 1).

The above-mentioned innovative study on complex properties of SBT, AR, and BC agro-waste and its derived components will allow replacing several chemically synthesized ingredients with natural ones with multi-functional properties in topical formulations. A cascading waste-free technological approach will provide additional income sources in rural areas, which is especially important in the agricultural sector off-season. Overall, it will make today’s fruit-shrubs-connected business based mostly on berries more economically feasible.

## 2. Results and Discussion

### 2.1. Chemical Composition of AR, SBT and BC Biomass and Changes in Oxidative Stability of LBS in Its Presence

#### 2.1.1. Analytical Pyrolysis Data

**Figure 2:**
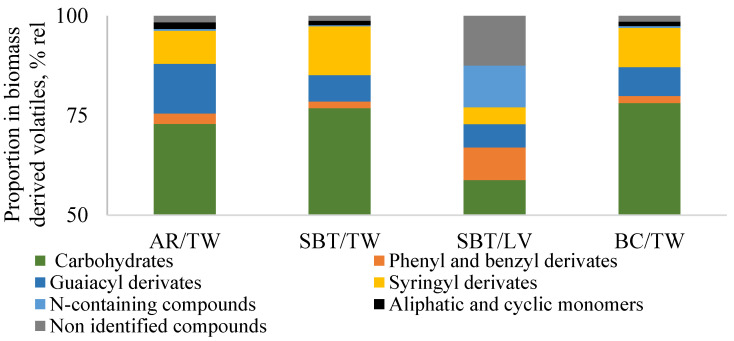
Analytical pyrolysis (Py-GC/MS/FID) data of SBT, AR, and BC twigs’ and SBT leaves’ biomass-derived volatiles. The variation coefficient of measurement was ≤5%.

The analytical pyrolysis Py-GC/MS/FID analysis reveals volatile substances originated from cellulose, hemicellulose, lignin, proteins, and secondary metabolites (extractives). Carbohydrates-derived volatiles (arose from aliphatic acids and esters, aliphatic aldehydes, ketones, aliphatic alcohols, pyran and furan derivatives, cyclopentane derivatives, and sugars) amounted to 73–78% of SBT, AR, and BC total twigs’ biomass and 59% of SBT/LV biomass volatile products (TVP) (Figure 2).

Nitrogen-containing volatiles of twigs and leaves confirmed the presence of nitrogen-containing compounds including proteins, which in SBT/LV were 10 times more than in the twigs.

Using Py-GC-MS-FID analysis data, structural characteristics of lignins of the composition of twigs’ and leaves’ biomass associated with antioxidant and antimicrobial activity were characterized. Literature data on the content of lignin in SBT, AR, and BC twigs’ biomass, and the relative proportions of syringyl (S) and guaiacyl (G) derivatives in it determined by analytical pyrolysis were not found, thus, this study is the first analyzing SBT, AR and BC twigs’ biomass lignins. Lignin thermal decomposition starts with the breaking of weaker bonds (α-ether and β-ether bonds), with a releasing of a mixture of methoxylated phenol, G, and S-type derivatives. The total lignin-derived volatiles content in SBT/LV and all twigs composition ranged from 10% to 21%/TVP that is within the usual range for deciduous trees. In comparison to leaves, the SBT/TW twigs had 1.9 times higher content of lignin-related G and S-type phenols. The content of lignin-related G and S-type phenols in all twigs samples was 6.6–12.5% TVP and 8.3–12.3% TVP, respectively.

It is known that the presence of ortho-methoxyl groups (G and S) has a positive effect associated with antioxidant activity [48,49,50,51,52]. The amount of G + S products describes the amount of phenylpropane units containing –O-CH_3_ groups. The difference between S/G proportion in leaves and twigs is statistically insignificant. Phenylpropane units in SBT leaves’ TVP is 10%, which is almost half as much as in twigs (17–21%). This indicates a higher antioxidant potential of the twig’s plant materials. However, the readily available phenols (represented by phenyl and benzyl derivatives detected by analytical pyrolysis) content in leaves (8%TVP) is higher than in twigs (2% TVP for SBT/TW), which corresponds to the literature data [53] indicating the additional antioxidant effect of this biomass. It could be said that biomass types are potent antioxidants. Since aging and inflammatory processes are commonly related to oxidative stress, it could be proposed that fruit trees’ lignocellulosic biomass have the potential as multifunctional anti-aging, anti-inflammatory as well as LBS system oxidation preventing component for creams, but the activity will depend also on the solubility in LBS [54]. According to the analytical pyrolysis data, the relative proportion of the twig biomass-derived volatiles had an insignificant difference between species in the relative proportion of chemical constituents considering CI (analytical pyrolysis data, Section 3.3.). The relative proportion of chemical components in SBT/LV differed more significantly from twigs, so leaves of SBT were further investigated as antioxidants of lipid systems (Section 2.1.2.) as well.

#### 2.1.2. Influence of Ground Biomass on the Oxidative Stability of the Lipid-Based System (LBS)

It can be seen that leaves samples better prevent lipid oxidation in the LBS with a higher content of lipids, but twigs samples—in the LBS system with lower lipids content (Table 1). It could be connected with a bigger content of lipophilic compounds in the leaves that have a better affinity to the system with higher lipids content. Hydrophilic compounds of the twigs have better solubility in the system with lower lipids content.

With the addition of 4% antioxidant (SBT/TW, SBT/LV, BC/TW or AR/TW), the protection factor of the cream further increased only 1.1–1.2 times in comparison with 2% of antioxidant on LBS, which indicates a supersaturation of the lipophilic and hydrophilic solution in the cream composition with the active components from biomass. Thus, according to Oxypress data, the SBT/TW and AR/TW work well as antioxidants, and in the amount of 2%/LBS increased the protection of the cream base with 19% lipid content from oxidation by 1.5–1.7 times compared to the control. SBT/LV in the amount of 2%/LBS increased the protection factor of the cream base by 1.4 (for the cream base with 19% lipid content) and 1.7 (for the cream base with 35% lipid content), respectively. Since further addition of the biomass gives less effect on the further increase in the oxidative stability, it seems that the most reasonable concentration of the biomass is around 2%. Further on, it is necessary to test the influence of the concentration of the stabilizer on the stability of the cream emulsion system.

To study possibilities to increase antioxidant activity, lipophilic and hydrophilic extracts of biomass were further evaluated.

### 2.2. Chemical Characterization of Lipophilic Extracts and Their Influence on the Oxidative Stability of the LBS

#### 2.2.1. Yield and Chemical Composition of the Lipophilic Extracts

Lipophilic extracts yield obtained by hexane from SBT/TW, AR/TW, and BC/TW biomass was very low (1.1–1.4% per DB) and it is clear that it is economically unreasonable to offer them as an antioxidant in cream formulations. However, lipophilic compounds as active nutrients could be further evaluated for improving overall skin health since they contain valuable active compounds. For example, linolenic acid participates in the formation of vitamin F, providing structure and flexibility to the outer layer of the cells, and helping with reducing the negative effect of UV radiation, which is important for aging skin [55,56]. The yield of lipophilic extract obtained by hexane from SBT/LV was 3.4%/DB.

Ozone-friendly hydrofluorocarbon (R134a, 1,1,1,2-Tetrafluoroethane) and hexane were compared as solvents on the example of SBT/LV. The yield of lipophilic extracts from SBT/LV was 2.7% and 3.4%, correspondingly (CI ≤ 0.7% and 0.4% at α = 0.05 for extraction by hydrofluorocarbon and hexane, correspondingly). The difference between the hydrofluorocarbon and hexane yield of lipophilic compounds is statistically significant. Testing of SBT lipophilic extracts from leaves on lipid oxidation was conducted assuming the multi-functionality of these ingredients.

The composition of lipophilic extracts of SBT/LV obtained by hydrofluorocarbon and hexane was evaluated by GC/MS/FID (Table 2).

The differences in the composition of the extracts could influence lipophilic extract activity in the LBS (Section 2.2.2).

The presence of phthalates, especially in the hexane extract that was taken for comparison, is most likely connected with the ability of hexane to extract plasticizers (such as phthalates) from plastic and rubber materials it contacts. That is why its avoiding in EU is the right decision. Some amount of phthalates could also be taken by plants from soil, but it is less possible in this case, since the plants were grown under conditions of minimal anthropogenic disturbance. Anyway, before application of the lipophilic extracts, preliminary GC/MS analysis is necessary and purification would be desirable, for example, as it described by Chen et al. [57], and it needs further investigation. The hydrofluorocarbon extract contains linolenic acid, an essential fatty acid for the health of cell membranes, which can have anti-aging and anti-inflammatory properties in skin care products. It was reported to have a role in repairing the skin barrier, improving wound healing and photoprotection [58]. 2-Propenoic acid could increase the viscosity of the cream and thus improve its texture. Erucic Acid can make skin softer and smoother, and it is beneficial for dry and aging skin. The most often-used source of it is jojoba oil [59]. Phytol is not only a fragrance-providing ingredient but has skin moisturizing and smoothing properties. Cyclopentadecanol is used in cosmetic products as fragrance, with proven absence of toxic effects in small dosages [60]. All these compounds’ effects and their allowed limits of concentrations could be further studied, but it is clear that adding lipophilic extracts could have complex beneficial effects on cream composition.

#### 2.2.2. Influence of Lipophilic Extracts on the Oxidative Stability of the LBS

Tests with the lipophilic extracts of the SBT/LV showed that only the ones obtained by hydrofluorocarbon improved the oxidative stability of cream with a lipid content of 35% in a concentration of 2% on LBS (Table 3).

This could be explained by the relative content of easily oxidized components in the hexane extract which was higher (55.7% rel/DE) than in the hydrofluorocarbon extract (30.3% rel/DE); the hydrofluorocarbon extract also contained a greater amount of aliphatic and cyclic monomers (43.1% rel/DE) (Table 2). However, several following factors have to be considered. 1,1,1,2-Tetrafluoroethane (hydrofluorocarbon R134a) has insignificant ozone depletion potential and negligible acidification (acid rain) potential. At the same time, its global warming potential exceeds the regulations established in 2021, and after January 2030 it has to be replaced by or mixed with the newer 2,3,3,3-Tetrafluoropropene R1234yf, which is much more expensive. But hexane is a toxic solvent that, after a while, can be banned for application in the EU. Therefore, since the effect of the lipophilic extract on the oxidative stability of the LBS is comparatively low, it is preferable to use biomass without extraction, or its hydrophilic extracts. The lipophilic extract can be validated for other potential benefits in cream composition.

### 2.3. Chemical Characterization of Hydrophilic Extract and PACs, and Their Influence on the Oxidative Stability of the LBS

#### 2.3.1. Yield and Chemical Composition of the Hydrophilic Extracts

Hydrophilic extracts obtained from twigs’ biomass, using distilled water as a solvent, had a yield varied from 14.0 to 15.0% per DB, correspondingly (CI ≤ 0.3%/DB). Using 50% EtOH as a solvent, the yield was bigger: AR/TW 17.8% > SBT/TW 15.5 > BC/TW 15.1%/DB, which pointed to the extractives better solubility in the ethanol-water solution. The content of polyphenols in the extracts of AR/TW and SBT/TW, obtained with 50% EtOH was the highest (48.6 and 46.2 g GAE·100 g^−1^ DE, correspondingly). The highest yield of hydrophilic extract was from SBT/LV—19.2%/DB (water extract) and 22.4%/DB (EtOH extract), but the content of polyphenols in the leaves was 2 times lower (22.6 g GAE·100 g^−1^ DE). According to Porter’s analysis data, oligomeric polyphenols (proanthocyanidins, PACs) are dominant polyphenolic compounds in the composition of hydrophilic extracts. These oligomeric polyphenols are recognized to be active metabolites of cellular metabolism and to be crucial to a number of plant physiological functions; PACs protect plants from pathogenic microbial attack, mechanical damage, and infectious illnesses. They also make trees more resistant to decay. The content of PACs of hydrophilic extracts obtained with 50% EtOH was more than 2 times higher in AR/TW (74.0%) than in the other twigs’ biomass (Table 4).

According to UHPLC-MS/MS data, oligomeric PACs of hydrophilic extracts was mainly composed of dimer, trimer and tetramer of B-type procyanidin, and procyanidin glycoside (Table 5). Compared to polymeric PACs, oligomeric PACs are more stable (pH 2.0–6.0) and more resistant to heat and light [61].

The compounds in Table 5 found in hydrophilic extracts are known for their beneficial properties in skin care. Low molecular weight polyphenolic compounds, such as catechin/epicatechin, quinic acid, gallic acid, salicylic acid, rutin, myricetin, quercetin, kaempferol, chlorogenic acid were present in all extracts. These compounds demonstrate a wide range of properties important for aging skin and medicinal topical formulations. For example, quercetin downregulates inflammatory signaling pathways, reduces inflammatory cell infiltration, production of pro-inflammatory cytokines, while increasing anti-inflammatory ones, suppresses IL-4 cytokines connected with chronic inflammation and atopic dermatitis, and thus supports wound healing [62]. Chlorogenic acid increases collagen synthesis in fibroblasts and improves skin’s barrier function [63]. Gluconic acid supports hydration—holds moisture in the skin and has antioxidant properties for protecting skin from environmental factors, e.g., UV radiation; supports the skin barrier and minimizes irritation; prevents hyperpigmentation and minimizes dark spots [64], an important property for aging skin. Catechins are well known for their anti-oxidative activity, ability to protect skin extracellular matrix, by promoting collagen synthesis and reducing expression of collagen degrading matrix metalloproteinases [65]. Rutin complements beforementioned skin anti-aging activities by promoting collagen production and thus increasing skin elasticity [66].

Based on the chemical characterization results (Figure 2 and Table 4 and Table 5) and the influence of the biomass on the oxidative stability of the LBS, twigs, and leaves can be considered a potential ingredient to protect the medicinal and cosmetic creams from oxidation, while also positively influencing skin health.

#### 2.3.2. Evaluation of the Influence of Hydrophilic Extracts and Purified PACs on LBS Stability

The results of DPPH and ABTS^+^ radical scavenging activity showed that all biomass PACs isolated from 50% EtOH extracts showed similar antioxidant activity (IC_50_ in average 2.4 mg·L^−1^ by DPPH· assay, and IC_50_ = 1.1 mg·L^−1^ by ABTS^+·^ assay) which was higher than that of Trolox (IC_50_ = 4.6 mg·L^−1^ by DPPH· assay and IC_50_ = 4.0 mg·L^−1^ by ABTS^+·^ assay), CI ≤ 0.3 mg·L^−1^ at α = 0.05. The hydrophilic extracts from AR/TW had the highest antioxidative activity among all extracts (IC_50_ = 3.8 mg·L^−1^ by DPPH· assay and IC_50_ = 2.6 mg·L^−1^ by ABTS^+·^ assay), but it was less than that of PACs.

The oxidative stability tests showed that at the same concentration (2%/LBS), the efficiency of hydrophilic extracts was 2.3–2.5 times higher for a cream with a lipid content of 19%) and 1.3–1.5 times higher for a cream with a lipid content of 35% than for biomass.

Although the antioxidant activity of purified PACs in tests with DPPH and ABTS^+^ radicals was much better than for the extracts, it was found that the influence of PACs on the oxidative stability of LBS is similar or less than for hydrophilic extracts. It could be connected with that the purified PACs dissolve in the lipid system only partially. Moreover, the activity of the extracts may be linked to more than just the presence of polyphenols, which are thought to be the most potent substances with antioxidant qualities, but with the combined action of low and high molecular polyphenols, and depending on their solubility in the system. Nevertheless, the Pearson’s correlation coefficient between LBS oxidation stability and PAC amount in the cream composition (introduced as a PACs-containing extract) showed positive correlation (r = 0.85).

Among hydrophilic extracts, AR had the highest effect on LBS stability. Compared to ascorbic acid, gallic acid, and tert-butylhydroquinone (TBHQ), all the studied extracts protected the creams from oxidation more effectively in the LBS with a lipid content of 35% (Table 6).

Comparing the data on antioxidant activity in cream with 35% and 19% lipid content, the extracts work better in a system with a lower lipid concentration. Considering the costs of PACs separation, it seems more reasonable to use the extracts without further PACs purification to stabilize LBS.

### 2.4. Evaluation of Extracts Antimicrobial Activity

To prove multi-functionality of waste lignocellulosic biomass of AR, SBT and BC, biomass extracts and PACs were evaluated as agents against bacteria that most often appear in creams, as well as bacteria causing a range of skin problems and acne (Table 7).

Purified PACs and 50% EtOH extracts showed better antimicrobial properties than water extracts. SBT 50% EtOH extract showed the best results for *P. aeruginosa*, *S. aureus*, and *B. cereus* among all biomasses. AR/TW extract was the most effective against *S. pyogenes* and *C. acnes*.

Earlier it was shown that SBT-derived PACs and extracts have anti-inflammation properties [26]. Clinical trials described in the literature confirmed the ability of PAC extract obtained from SBT berries to support stem cell types involved in regenerative and reparative functions [71]. More studies showed AR berries anti-inflammatory, hypotensive, antiviral, and anticancer properties [72], and the ability of BC berries anthocyanins to increase the level of collagen [73]. Thus, the waste biomass of SBT, AR, and BC could be further studied for the same properties. The findings support the concept of the application of multi-purpose ingredients based on SBT/TW, AR/TW, and BC/TW extracts and biomass-derived PACs in topical formulations. One or the other ingredient and biomass source can be chosen depending on the target properties of the cream formulation.

### 2.5. Evaluation of the Emulsion Stability and pH Tests of the Creams with Extracts

The tests with dry extracts made according to the methodology described in Section 3.10 showed no negative influence on the emulsion stability which is one of the important characteristics of the cream quality. pH of the creams with the addition of the extracts were 5.2–5.5, within the normal human skin acidic range (pH 4.5–6.5, in average 5.5).

The results were consistent with the available literature data for the fruits polyphenols obtained from grapes [74] and proved that the extracts can be incorporated into creams as a multifunctional components without compromising their quality.

### 2.6. Cytotoxicity and Phototoxicity of PACs and Hydrophilic Extracts

**Figure 3:**
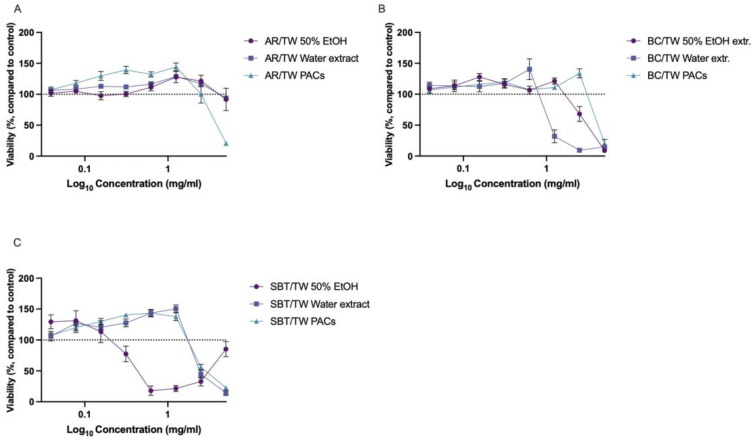
Cytotoxicity of extracts and PACs from AR/TW (**A**), BC/TW (**B**) and SBT/TW (**C**). Results expressed as changes in viability compared to control cells. Dotted line indicates average control level (100%), *n* = 6.

Cytotoxicity testing showed that AR/TW extracts had no negative effect on keratinocyte viability throughout the concentration of 0.039–5 mg·mL^−1^, while PACs exhibited cytotoxic activity only at the highest tested concentration. Interestingly, within the range of 0.156–1.25 mg·mL^−1^, AR/TW PACs stimulated cell proliferation, as evidenced by increased viable cell counts compared with the control. These differences were statistically significant from the control (*p* < 0.001, one-way ANOVA). A similar but less pronounced stimulating effect was also observed for the water and 50% ethanol extracts (Figure 3A). Our results are in line with previously reported HaCaT proliferation stimulating effect of aronia extracts [75]. The blackcurrant PACs produced a cytotoxic effect only at the highest tested concentration (5 mg·mL^−1^), while the 50% ethanol extract was cytotoxic at 2.5 mg·mL^−1^ and the water extract at 1.25 mg·mL^−1^ (Figure 3B). The effects of the SBT/TW water extract and PACs were similar, while the SBT/TW 50% ethanol extract demonstrated a cytotoxic effect at concentrations of 0.313 and above. Within the concentration range of 0.313–1.25 mg·mL^−1^, the SBT/TW water extract and PACs demonstrated a cell proliferation-stimulating effect that differed statistically significantly from the control (one-way ANOVA, *p* < 0.001) (Figure 3C). Differences in the effects of the SBT/TW extracts and PACs indicate that most probably the ethanolic extracts contain a higher total amount of polyphenols and could have higher concentrations of compounds that inhibit keratinocyte proliferation, for example, kaempferol [76]. This phenomenon demands further investigation, which could also be useful for the study of the extract’s activity in psoriasis treatment.

**Figure 4:**
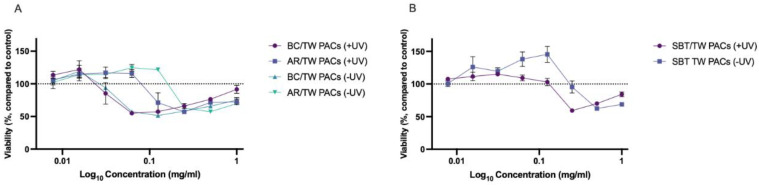
Viability of HaCaT keratinocytes in phototoxicity assay after pre-incubation with PACs from BC/TW and PACs from AR/TW (**A**) and PACs from SB/TW (**B**) followed by irradiation with 5 J/cm^2^ UVA. +UV—UV-irradiated keratinocytes after pre-incubation with PACs, −UV—non-irradiated keratinocytes with PACs. Dotted line indicates average control level (100%) *n* = 3.

The results of the phototoxicity assays reveal that the tested PACs and hydrophilic extracts exhibit mild phototoxicity. Changes in the viability of the HaCaT keratinocytes pre-incubated with BC/TW PACs was independent of UV treatment, while in AR/TW PACs and SBT/TW PACs, keratinocyte pre-incubation before UV treatment slightly reduced cell viability compared to non-irradiated cells (Figure 4).

No statistically significant differences in cell viability between non-irradiated and UV-irradiated keratinocytes after pre-incubation with PACs were observed. Interestingly, AR/TW PACs and SBT/TW PACs had a slightly positive effect on cell viability and proliferation both in non-irradiated and UV-treated keratinocytes at concentrations below 0.063 mg·mL^−1^. Over the concentration range of 0.016–0.063 mg·mL^−1^, AR/TW PACs in non-irradiated cells increased cell viability by an average of 17.42 ± 6.0%, and in UV-irradiated cells by 15.79 ± 0.97%. In the concentration range of 0.016–0.125, SBT/TW PACs increased cell viability by 32.24 ± 11.63% in non-irradiated cells, but in UV-treated cells, a slight increase of 12.0 ± 2.94% was observed at concentrations of 0.016–0.063 mg·mL^−1^.

Non-phototoxic (cell viability above 80%) concentrations for BC/TW PACs were determined to be ≤0.03 mg·mL^−1^, for AR/TW PACs—≤0.063 mg·mL^−1^, and for SBT/TW PACs—≤0.125 mg·mL^−1^.

It should be emphasized that non-phototoxic concentrations of AR/TW and BC/TW PACs are lower than most of the determined MIC concentrations against skin pathogens, whereas SBT/TW PACs are the least phototoxic. Additionally, the MIC values of SBT/TW PACs against several skin pathogens are well below the upper non-phototoxic concentration.

**Figure 5:**
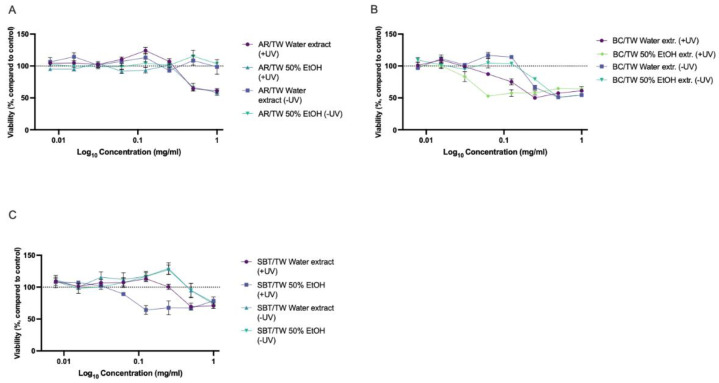
Viability of HaCaT keratinocytes in phototoxicity assay after pre-incubation with AR/TW extracts (**A**), BC/TW extracts (**B**), and SBT/TW extracts (**C**), followed by irradiation with 5 J cm^−2^ UVA. +UV—UV-irradiated keratinocytes after pre-incubation with extracts, −UV—non-irradiated keratinocytes with extracts. Results expressed as changes in cell viability (%) compared to corresponding solvent controls. Dotted line indicates average control level (100%), *n* = 3.

For topically used products and their ingredients, evaluation of phototoxicity is important. Phototoxicity evaluation of the extracts revealed that AR/TW extracts, independent of the solvent used, had the least negative effect. AR/TW extracts in non-irradiated keratinocytes did not change cell viability at any of the tested concentrations. Under UV treatment concentrations of 0.5 and 1.0 mg·mL^−1^ decreased cell viability below 65% (Figure 5A). Non-phototoxic concentrations of both AR/TW extracts were determined to be ≤0.25 mg·mL^−1^. Independent of the solvent used, both AR/TW extracts showed phototoxicity only at concentrations higher than those needed for growth inhibition of *B. cereus* and *S. pyogenes*, and in case of AR/TW water extract also lower than MIC for *S. aureus*.

The BC/TW water and 50% ethanol extracts showed phototoxic effects in HaCaT keratinocytes in the concentration range of 0.031–0.125 mg·mL^−1^; however, the differences in the viability of UV-treated and non-irradiated cells were not statistically significant (Figure 5B). At concentrations below 0.031 mg·mL^−1^ and above 0.125 mg·mL^−1^, viability of the keratinocytes was similar in irradiated and non-irradiated cells. UV irradiation induced more pronounced reduction in cell viability in keratinocytes pre-incubated with 50% ethanol extract. In the concentration range of 0.063–1 mg·mL^−1^, cell viability dropped below 65%. The BC/TW water extract showed a slight stimulating effect at concentrations 0.063 and 0.125 mg·mL^−1^. The non-phototoxic concentration of the BC/TW water extract was ≤0.063 mg·mL^−1^, and the BC/TW 50% ethanol extract was ≤0.031 mg·mL^−1^. SBT/TW extracts in non-irradiated HaCaT cells had a negative effect on cell viability only at the highest tested concentration (1 mg·mL^−1^). A phototoxic effect at concentrations 0.125 mg·mL^−1^ and above was observed in the case of the SBT/TW 50% ethanol extract, while the water extract reduced the cell viability of UV-treated keratinocytes only at concentrations of 0.5 mg·mL^−1^ and 1 mg·mL^−1^ (Figure 5C). Non-phototoxic concentrations of SB/TW 50% ethanol extract were ≤0.031 mg·mL^−1^, and the SBT/TW water extract was ≤0.125 mg·mL^−1^.

Even though extracts did not show pronounced phototoxicity at low concentrations, differences between extract sources and the extraction solvents used should still be considered when selecting materials for topical formulations, especially if the topical product is intended for use before sun exposure.

## 3. Materials and Methods

### 3.1. Reagents

The high purity solvents, stable free radicals 2,2-diphenyl-1-picrylhydrazyl (DPPH·) and 2,2′-azinobis(3-ethylbenzothiazoline-6-sulfonic acid) (ABTS^+·^); analytical standards procyanidin B2 (purity ≥ 90%), gallic acid (purity ≥ 97.5%), and ascorbic acid (purity 99%), as well as the reference antioxidant Trolox (6-hydroxy-2,5,7,8-tetramethylchroman-2-carboxylic acid) were acquired from Sigma-Aldrich (Sigma-aldrich, Inc., St. Louis, MO, USA). Methanol (purity ≥ 99.9%, suitable for HPLC) was purchased from Honeywell Fluka™ (Honeywell Fluka, Seelze, Germany); and formic acid, ACS reagent, purity ≥ 98%—from Sigma-Aldrich (Sigma-aldrich, Inc., St. Louis, MO, USA). Stackpure purification system (OmniaTap 6, Niederahr, Germany) was used for preparation of the Type 1 ultrapure water, for the mobile phase and sample preparation. All solutions were degassed by sonication for 30 min. Reference analytical standards for quantitative analyses were purchased: (+)-catechin analytical standard, HPLC purity ≥ 99.0%, from Supelco (Supelco, Buchs, Switzerland); (-)-epicatechin, HPLC purity ≥ 98.0%, from Sigma-Aldrich (Sigma-aldrich, Inc., St. Louis, MO, USA); (-)-epigallocatechin, HPLC purity ≥ 95%, from Sigma-Aldrich (Sigma-aldrich, Inc., St. Louis, MO, USA); quercetin, HPLC purity ≥ 95%, from Sigma-Aldrich (St. Louis, MO, USA); kaempferol, HPLC purity ≥ 97.0%, from Sigma-Aldrich (Sigma-aldrich, Inc., St. Louis, MO, USA); caffeic acid, HPLC purity ≥ 98.0%, from Sigma-Aldrich (Sigma-aldrich, Inc., St. Louis, MO, USA); myricetin, HPLC purity ≥ 96.0%, from Sigma-Aldrich (Sigma-aldrich, Inc., St. Louis, MO, USA); p-coumaric acid, HPLC purity ≥ 98.0%, from Sigma-Aldrich (Sigma-aldrich, Inc., St. Louis, MO, USA); rutin, HPLC purity ≥ 95.0%, from PhytoLab (PhytoLab GmbH & Co. KG, Vestenbergsgreuth, Germany).

Reference microbial strains: *C. acnes* MSCL 1521, *S. aureus* MSCL 3340, *P. aeruginosa* MSCL 3314, *B. cereus* MSCL 330, *S. pyogenes* MSCL 620 were received from the Microbial Strain Collection of Latvia (MSCL, Riga, Latvia). Cosmetic and medicinal cream bases (with 19% and 35% lipid content, correspondingly) without added antioxidants were received from private companies producing cosmetic creams and medicinal topical formulations.

HaCaT human keratinocytes were purchased from Cell Lines Services, Germany. Cell cultivation media and reagents for phototoxicity assay: DMEM—Dulbecco’s modified Eagle’s medium (Sigma-aldrich, Inc., St. Louis, MO, USA), fetal bovine serum (FBS, Sigma-Aldrich Inc., St. Louis, MO, USA), streptomycin/penicillin solution (Sigma-Aldritch, St. Louis, MO, USA), neutral red dye (Sigma-aldrich, Inc., St. Louis, MO, USA), phosphate-buffered saline (PBS) (Sigma-aldrich, Inc., St. Louis, MO, USA), dimethyl sulfoxide (DMSO, Sigma-aldrich, Inc., St. Louis, MO, USA), glacial acetic acid (Sigma-aldrich, Inc., St. Louis, MO, USA).

### 3.2. Collection of SBT, AR, BC Plant Material

The SBT leaves and twigs plant material (LV and TW, correspondingly) were obtained from the SBT plantation in Tukums county, near Engure, Latvia (DD: 57.1444093, 23.108156), at the end of summer of 2023. Both LV and TW (after separating the berries and LV), were dried at room temperature. AR and BC TW plant material was obtained from the Baldone area’s fruit tree/shrub plantation in, Kekava county, Latvia (DD: 56.82065, 24.27653), in the beginning of autumn of 2023.

Dried TWs were thermally treated by hot water and ground with Retsch SM100 knife mill (Retsch GmbH, Haan, Germany), to the 2–4 mm particle size. The SBT leaves were ground with the same knife mill to obtain a particle size of 1–2 mm.

### 3.3. Chemical Characterization of Biomass

#### Analysis by Analytical Pyrolysis (Py-GC/MS/FID)

Analytical pyrolysis was carried out using Pyroliser Frontier Lab Micro Double-shot Py-2020iD (temperature of pyrolysis was 500 °C, rate of heating 600 °C s^−1^) coupled directly with Shimadzu gas chromatograph-mass spectrometer GC/MS/FID-QP ULTRA 2010 (Shimadzu, Kyoto, Japan), and applying a RTX-1701 capillary column (Restec, Metairie, LA, USA), as described by Grillo et al. [77]. Library MS NIST 11 and NIST 11s were used to identify each unique compound. The peak relative area was calculated using Shimadzu software NIST version 5 as described by Alves [78]. The relevant peaks’ summed molar areas were normalized to 100%. The evaluations were repeated four times for each sample. The variation coefficient of the measurement was ≤5%.

### 3.4. Preparation of Biomass Extracts and PACs

Lipophilic extracts of LV and TW biomass were isolated in two ways: (1) by hexane, heating temperature 50 °C, heating time 30 min; (2) by 1,1,1,2-tetrafluoroethane (hydrofluorocarbon R134a) at the temperature 17–20 °C, multiple cycles, extraction time 24 h. Hydrophilic extracts were obtained with distilled water, and ethanol-water solutions (*v*/*v* 50:50), extraction temperature 60 °C, time 30 min. Extracts were lyophilized by freeze-drying at −55 °C using Heto Power Dry PL3000 equipment (Thermo Fischer Scientific, Waltham, MA, USA). The yield is given in % on dry biomass (DB). Confidence interval (CI) ≤ 0.7% at α = 0.05.

For separation of PACs from non-PACs compounds and sugar a separation column was used, filled with solvent-resistant size exclusion crosslinked resin Sephadex™ LH-20, and 96% EtOH and 70% (*v*/*v*) acetone as eluents. Phenolic compounds of low-molecular weight were separated using 96% EtOH, PACs using 70% (*v*/*v*) acetone. Solvent was evaporated in a Heidolph Hei-VAP Core glassware core set G3 rotary evaporator All-round Chill Package (Heidolph Instruments, Schwabach, Germany), and purified PACs before were freeze-dried. The samples were stored at −8 °C.

### 3.5. Chemical Characterization of Lipophilic Extracts by GC/MC/FID Analysis

Analysis of lipophilic extracts was conducted by gas chromatography-mass spectrometry, GC/MS/FID-QP ULTRA 2010 equipment (Shimadzu, Kyoto, Japan), applying 60 m RTX-1701 capillary column (Restec, Metairie, LA, USA); mass spectrometer electron impact mode 70 eV, injector t = 250 °C, with helium as a carrier gas (1 mL min^−1^), a split ratio 1:30. Extracts with a moisture less than 1% were solved in hexane (*w*/*w* 1:10), filtered using filter with 0.45 μm pores. Oven heating was conducted for 1 min isothermally at 60 °C, then to 270 °C, rate 6 °C min^−1^, then keeping 10 min at 270 °C. Individual compounds were interpreted according to the MS NIST 11 and NIST 11s Libraries.

### 3.6. Chemical Characterization of Hydrophilic Extracts

#### 3.6.1. Determination of Content of Total Polyphenols in the Extracts

The Folin–Ciocalteu method was used to measure the content of total polyphenols (TP) in the hydrophilic dry extracts (DE). Gallic acid was used as a reference compound as described by Chen et al. [79] (standard calibration curve is in the Supplementary Materials, Figure S3). The results were given as g of gallic acid per 100 g of DE (g GAE·100 g^−1^ DE). Each extract was analyzed in triplicate. CI ≤ 0.4 g GAE·100 g^−1^ DE, at α = 0.05.

#### 3.6.2. PACs Content Determination in Hydrophilic Extracts and Purified PACs Samples

The analysis of the PAC amount in the hydrophilic extracts as well as in the samples of purified PACs was performed by the butanol-HCl method. Procyanidin dimer B2 standard was used as a reference [25]. The PAC amount was given on DE as a percentage. Three repetitions were conducted for each sample. CI ≤ 0.4% at α = 0.05.

A standard calibration curve was generated for 7 concentrations: 0.004 mg·mL^−1^, 0.025 mg·mL^−1^, 0.05 mg·mL^−1^, 0.1 mg·mL^−1^, 0.25 mg·mL^−1^, 0.5 mg·mL^−1^ and 1 mg·mL^−1^ (Figure S4 in Supplementary Materials).

#### 3.6.3. Extracts Qualitative Analysis by Liquid Chromatography-Mass Spectrometry (LC-MS)

The liquid chromatography UHPLC-MS/MS method applying Vanquish Flex UHPLC system (Thermo Fisher Scientific, Germering, Germany), was used for analysis, with a Binary Pump F, Split Sampler FT, and using a Zorbax Eclipse Plus C18 column (2.1 × 150 mm, 5 μm (Agilent, Santa Clara, CA, USA). The A mobile phase was 0.1% formic acid solved in ultrapure water, but B mobile phase was 0.1% formic acid solved in methanol. The mobile phase flow rate was 0.4 mL min^−1^, the temperature of column was maintained 40 °C. The gradient was the following: 0 min, 5% B; 1.0 min, 5% B; 15.0 min, 30% B; 20.0 min, 50% B; 25.0 min, 70% B; 26.0 min, 95% B; 28.0 min, 95% B; 29.0 min, 5% B; and 30.0 min, 5% B. Time of equilibration: 3 min; injection volume: 1 μL. High- resolution mass spectrometry was performed in Orbitrap LS-MS Exploris™ 120 (Thermo Fisher Scientific, Bremen, Germany) with Optamax NG Electrospray ion source a heated electrospray ionization (Thermo Scientific™ HESI-II) probe for enhanced sensitivity (Thermo Fisher Scientific, Inc., Waltham, MA, USA). Both negative and positive ionization modes were applied, the chosen S-lens RF level was 70%;the range: 100 *m*/*z* to 1500 *m*/*z*. The spray voltage for the mass spectrometer was 2.5 kV (−) and 3.5 kV (+);gas flow rates were 50 for sheath gas and 10 for the auxiliary gas; ion transfer tube temperature (temp) was maintained 325 °C, and the vaporizer temp 350 °C; full scan mode was applied (resolution 30,000) followed by data-dependent (dd) ddMS2 scans for selected ions (resolution 15,000), with absolute collision and HCD collision energies 15, 40, and 70. Xcalibur 4.6 instrument control and data handling software was used for data processing (Thermo Fisher Scientific, Waltham, MA, USA). UHPLC-HRMS spectra were analyzed with a help of TraceFinder 5.1 (Thermo Fisher Scientific, Waltham, MA, USA). A compounds database (110) was created for the identification of individual substances through a variety of literary sources and libraries, including PubChem, FoodDB, KNApSAcK, and mzCloud mass spectral fragmentation database.

### 3.7. Determination of the Radical Scavenging Activity

Radical scavenging activity of hydrophilic extracts and purified PACs was determined using the stable free radicals 2,2-diphenyl-1-picrylhydrazyl (DPPH·) and ABTS^+·^ (2,2′-azinobis(3-ethylbenzothiazoline-6-sulfonic acid). The DPPH· assay was measured and calculated in accordance with the procedures outlined by Dizhbite et al. [50]. CI ≤ 0.3 mg·L^−1^. Equation is in Supplementary Materials (Figure S5).

The ABTS^+·^ assay was performed as described in [80]. The absorption of different concentrations of solutions was measured utilizing spectrophotometer LAMBDA 650 UV/Vis (Perkin Elmer, Shelton, CT, USA). The measurements were conducted in triplicate, and presented antioxidant concentration, mg∙L^−1^, necessary for inhibition of 50% of the free radicals (IC_50_). A synthetic analog of vitamin E (Trolox) was used as a reference (Equation is in Supplementary Materials, Figure S6). CI ≤ 0.3 mg·L^−1^.

### 3.8. Oxidative Stability of the Lipid-Based System (LBS)

The influence of samples (biomass after thermal pre-treatment, biomass extracts, and PACs) on the LBS oxidation stability was studied with cosmetic cream and topical drug bases (basic composition without any added antioxidant), using Mikrolab Oxipress™ equipment (Mikrolab Aarhus, Højbjerg, Denmark). Content of the lipid phase in cosmetic and medicinal creams was 19% and 35%, correspondingly. Creams were kindly provided by “Madara Cosmetics” and “Magic You” (Latvia). The oxidative stability was determined under the optimum conditions described by Trojakova et al. [81], with slight modifications. The cream base was placed into reaction vessels in the amount corresponding to 5 g of the lipid phase (26.3 g and 14.3 g for 19% and 35% creams, correspondingly). The sample was thoroughly mixed with the cream for 10 min in the reaction vessel. The reaction vessel was connected to oxygen, the air was expelled and then oxygen was introduced to the vessel to the specified pressure, set as 0.5 MPa, and the temperature was set as 120 °C. The LBS without antioxidants was used as a control. Time-dependent variations in O_2_ pressure were recorded. The protection factor (PF) of the samples was determined according to Equation (1):

PF = IPx/IPc,(1)

**Figure 6:**
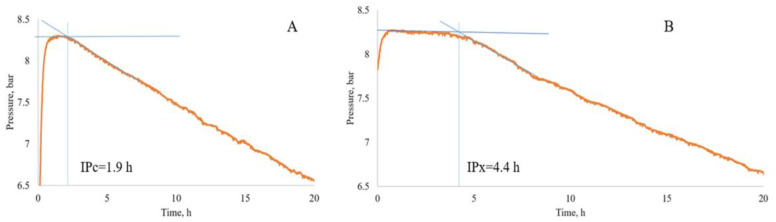
Influence of the sample on the oxidation time of cream (**A**)—cosmetic cream base without antioxidant (IPc–the induction period, h of cream oxidation without a sample); (**B**)—with the addition of the antioxidant sample into cosmetic cream (IPc–the induction period, h of cream oxidation when there is a sample).

In the equation, IPx and IPc: the induction period (time, h) of cream oxidation when there is a sample and one of the blank, respectively (Figure 6). CI is shown in the results section under the Tables.

### 3.9. Antimicrobial Activity Against Skin-Care Contaminating and in Sebum Developing Bacteria

Reference bacterial strains were obtained from the University of Latvia, Microbial Strain Collection of Latvia (MSCL): *C. acnes* MSCL 1521, *P. aeruginosa* MSCL 3314, *S. aureus* MSCL 3340, *B. cereus* MSCL 330, and *S. pyogenes* MSCL 620. The extracts’ and pure PACs’ antimicrobial activity was evaluated by determination of the microorganisms sensitivity to antimicrobial agents, in 96-well plates using twofold broth microdilution method, which outcomes are the minimum inhibitory (MIC) and minimum bactericidal concentrations (MBC) [82]. Incubation in 96-well plates was conducted at 37 °C, for *P. aeruginosa*, *S. aureus*, and *B. cereus*: 24 h, and for *S. pyogenes* and *C. acnes*: 48 h. Mueller-Hinton broth and agar were used for aerobic cultivation of *P. aeruginosa*, *S. aureus*, *B. cereus*, and *S. pyogenes*, and Wilkins-Chalgren Anaerobe broth and agar was used for anaerobic cultivation (BD GasPak EZ) of *C. acnes*. The results (MIC and MBC) expressed as median of 3 replicates.

### 3.10. Emulsion Stability and pH Tests

For evaluation of the emulsion stability after adding the extracts and PACs to the cream base, centrifuge Heraeus Biofuge Primo R (Thermo Scientific, Hanau, Germany) test was performed at 3000 rpm during half an hour, at room temperature (22 ± 1 °C), allowing to observe phase separation and sedimentation.

The pH values of the creams with added biomass, extracts and PACs were observed using a pH meter Hanna Instruments HI2002 (UAB HANNA Instruments Baltics, Vilnius, Lithuania) at room temperature (22 ± 1 °C). All measurements were repeated 3 times.

### 3.11. Cytotoxicity Assay

Cytotoxicity was tested in HaCaT human keratinocytes (Section 2.1). Testing was performed according to the description in OECD Guidance document No. 129 (2010) with slight modifications. The test samples were dissolved in dimethyl sulfoxide (DMSO, Sigma, Irvine UK) diluted with cell cultivation media and filtered prior to adding to the cell cultures. seeded 96-well microplates were used for cells seeding at a concentration of 8 × 10^3^ cells per well. After seeding plates were left to incubate overnight at 37 °C, 5% CO_2_ in standard cell cultivation media (DMEM with addition of fetal bovine serum (10%), penicillin/streptomycin (1%), (all from Sigma)). For washing the cells, PBS was used, and 100 μL of media mixed with test samples was added to the cells. Cell in media containing solvent at concentration corresponding to that in the test samples were used as reference. Following 48 h of incubation, the cells were washed with PBS, and 250 μL of 25 μg/mL Neutral Red dye solution in 5% serum containing media was added to every well; incubated 3 h at the same conditions and again washed with PBS. Neutral red dye was extracted by adding desorb solution (100 μL) which consisted of 50% ethanol, 49% water and 1% glacial acetic acid to every well. Plates we incubated with desorb solution for 30 min at room temperatures with mild shaking. Absorbance was measured using microplate reader Tecan M200 Infinite Pro (Tecan, Mannedorf, Switzerland) at 540 nm. Samples cytotoxicity was calculated as changes in cell viability relative to the solvent control according to the formula given below Equation (2):

Viability (%) = (Abs_540 (Sample) − Abs_540 (Background))/ (Abs_540 (control) − Abs_(540) (background)) × 100%(2)

### 3.12. Phototoxicity Tests

The in vitro phototoxicity assay was performed in HaCaT keratinocyte cell line according to slightly modified method described in OECD Test Guideline No. 432 (OECD 2019). 96-well microplates were used for cells seeding, concentration was 8 × 10^3^ cells per well. After overnight incubation at 37 °C, 5% CO_2_ in standard cultivation media cells were rinsed with PBS. Test samples were diluted in PBS, filtered and added to cells. Cells received treatment with test samples during one hour in dark in 96-well plates, then exposed to 5 J cm^−2^ UVA light using UVA CUBE 400 UV curing chamber (Honle AG, Gilching, Germany). In parallel, duplicate plates with cells were treated for one hour with the same samples without UVA irradiation at the end of incubation time. After UVA irradiation (for the UV treated plates) and at the end of dark incubation (for the UV-untreated plates) cells were rinsed with PBS, fresh standard cultivation media was added to every well, and they were incubated for 24 h. After incubation, Neutral Red dye uptake assay was performed, and changes in cell viability were assessed according to the procedure described in Section 3.11 “Cytotoxicity assay”.

### 3.13. Statistical Analysis

The experiments were conducted with 3 replications, except for analytical pyrolysis and gas chromatography (GC/MS/FID analyses, where experiments were conducted in 4 replications. The results were shown as mean values. Statistical analyses were conducted utilizing the Microsoft Corporation spreadsheet tool program Microsoft Excel 2016. Confidence intervals (CIs) for means were calculated using Student’s T distribution applying 5% (α = 0.05) significance level. Cytotoxicity and phototoxicity data were analyzed in GraphPad Prism 9.

## 4. Conclusions

The study confirmed that SBT, AR, and, to a lesser extent, BC lignocellulosic agro-waste after harvesting and pruning can be effectively used even after small pre-treatment in lipid-based systems, decreasing the oxidation rate from 1.3 to 4 times. Extracts of SBT, AR, and BC waste biomass and their derived proanthocyanidins are even more effective as LBS stabilizers and have antimicrobial properties against pathogenic bacteria that develop in sebum, pose problems for humans, and can contaminate skin care products (*C. acnes*, *P. aeruginosa*, *S. aureus, B. cereus*, and *S. pyogenes*). Along with the anti-inflammatory properties studied by authors earlier for SBT waste biomass and the confirmed presence of biologically active compounds described in the literature as anti-aging (e.g., chlorogenic acid, gluconic acid), anti-inflammatory (e.g., quercetin), and dryness-preventing (e.g., linoleic acid, erucic acid, etc.), the biomass of SBT, AR, and BC, its lipophilic and hydrophilic extracts, and PACs have potential for multipurpose applications in topical formulations, which can replace several chemically synthesized ingredients.

In vitro cytotoxicity and phototoxicity testing identified aronia twig extracts and their PACs as the safest among the extracts evaluated. The safety profiles of blackcurrant and sea buckthorn extracts and their PACs varied depending on the plant source and extraction method. This study highlights the importance of in vitro safety assessment as a complementary step to bioactivity testing and phytochemical characterization in the development of safe and effective topical products.

Moreover, since all the compounds are of plant origin, they are suitable for vegans, vegetarians, and people looking for an ethical choice of cosmetics. The wasteless SBT, AR, and BC cascading processing scheme offered in this study (fruit—for food and pruning waste—for cosmetic and medicinal creams) will help develop a sustainable economy based on the available natural resources in rural areas.

## Figures and Tables

**Table 1:** Influence of leaves’ and twigs’ biomass on the oxidative stability of creams.

Sample	IP *, h	PF **	IP *, h	PF **
	Lipid Content in Cream– 19%		Lipid Content in Cream–35%	
Cream base without antioxidants	11.0	1.0	1.9	1.0
Cream base + 1% BC/TW on LBS	13.2	1.2	1.9	1.0
Cream base + 2% BC/TW on LBS	14.6	1.3	2.2	1.2
Cream base + 4% BC/TW on LBS	14.6	1.3	2.4	1.3
Cream base + 1% AR/TW on LBS	14.1	1.3	2.1	1.1
Cream base + 2% AR/TW on LBS	18.2	1.7	2.6	1.2
Cream base + 4% AR/TW on LBS	20.1	1.8	2.8	1.5
Cream base + 2% SBT/TW on LBS	13.7	1.3	1.9	1.0
Cream base + 2% SBT/TW on LBS	16.8	1.5	2.1	1.1
Cream base + 4% SBT/TW on LBS	19.8	1.8	2.4	1.3
Cream base + 1% SBT/LV on LBS	13.2	1.2	3.2	1.7
Cream base + 2% SBT/LV on LBS	15.1	1.4	3.2	1.7
Cream base + 4% SBT/LV on LBS	16.8	1.5	3.2	1.7

The confidence interval for all the results: CI ≤ 0.2 at α = 0.05. * IP—the induction period (time, h) of cream oxidation when there is a sample in the cream; ** PF—the protection factor.

**Table 2:** GC/MS/FID data of identified compounds in lipophilic extract of SBT/LV composition isolated by hydrofluorocarbon and hexane.

Identified Compounds	Hydrofluorocarbon Extract, % rel	Hexane Extract, % rel
2-Propenoic acid, 2-methyl-, 1,4-butanediyl ester (4-(2-methylprop-2-enoyloxy)butyl 2-methylprop-2-enoate)	4.5	-
Linolenic acid ((9Z,12Z,15Z)-octadeca-9,12,15-trienoic acid)	2.1	-
Stearic Acid (octadecanoic acid)	2.4	1.9
Phthalic acid, diisobutyl ester (bis(2-methylpropyl) benzene-1,2-dicarboxylate)	0.6	1.0
Palmitic acid (hexadecanoic acid)		5.1
9,12-Octadecadienoic acid, methyl ester (methyl (9Z,12Z)-octadeca-9,12-dienoate)	1.5	1.5
Palmitoleic acid ((Z)-hexadec-9-enoic acid)		1.4
Phthalic acid, dibutyl ester (dibutyl benzene-1,2-dicarboxylate)	1.1	8.9
9-Octadecenoic acid, ethyl ester (ethyl (E)-octadec-9-enoate)	4.4	8.4
9,12-Octadecadienoic acid, methyl ester (methyl (9Z,12Z)-octadeca-9,12-dienoate)	-	3.5
Stearolic acid (octadec-9-ynoic acid)	-	1.7
Behenic acid (docosanoic acid)	-	1.5
(E)-13-Docosenoic acid ((E)-docos-13-enoic acid)	-	7.8
Tridecanedioic acid, diethyl ester (diethyl tridecanedioate)	-	5.2
Docosanoic acid, ethyl ester (ethyl docosanoate)	-	3.9
Erucic acid (13-Docosenoic acid, (Z) (Z)-docos-13-enoic acid)	2.2	1.9
Total acid/ester relative content in DE	18.8	53.7
1-butyl-2-ethylcyclobutane	0.5	-
Undecanal	0.8	-
Dodec-1-ene	2.4	-
Pentadec-1-ene	3.8	-
1-methyl-2-octylcyclopropane	0.7	-
Hexadec-1-ene	3.5	-
(E)-tridec-2-enal	2.3	-
(2E,6E)-3,7,11-Trimethyldodeca-2,6,10-trien-1-ol	-	23.7
Pentadecanal	1.5	0.9
Cyclopentadecanol	5.8	3.0
6,10,14-trimethylpentadecan-2-one	3.0	1.1
Octadec-1-ene	1.6	-
Phytol ((E,7R,11R)-3,7,11,15-tetramethylhexadec-2-en-1-ol)	6.5	-
(Z)-pentadec-5-en-7-yne	3.4	-
5-methyl-5-(4,8,12-trimethyltridecyl)oxolan-2-one	7.3	1.2
Total aliphatic and cyclic monomers in DE	43.1	29.9

**Table 3:** SBT/LV lipophilic extract influence on the oxidative stability of creams.

Sample	IP *, h	PF **	IP, h	PF
	Lipid Content in Cream–19%		Lipid Content in Cream–35%	
Cream base without antioxidants	11.0	1.0	1.9	1.0
Cream + 1% SBT/LV/FR *** on LBS	11.1	1.0	2.2	1.2
Cream + 2% SBT/LV/FR *** on LBS	11.6	1.1	3.5	1.8
Cream + 2% SBT/LV/HX **** on LBS	11.4	1.0	2.2	1.2

* IP—the induction period (time, h) of cream oxidation when there is a sample in the cream; ** PF—the protection factor. *** SBT/LV/FR lipophilic extract obtained by hydrofluorocarbon from SBT/LV. **** SBT/LV/HX lipophilic extract obtained by hexane from SBT/LV. The confidence interval for all the results did not exceed 0.2 at α = 0.05.

**Table 4:** Chemical characterization of the hydrophilic extracts from twigs’ and leaves’ biomass.

Sample	Yield of Extract from Biomass, %/DB	Total Polyphenols Content in Extract, g GAE·100 g^−1^ DE	Content of PACs in Extract, %/DE
Water extracts			
SBT/TW	14.4 ± 0.1	26.2 ± 0.2	16.4 ± 0.1
SBT/LV	19.2 ± 0.1	24.2 ± 0.1	0
BC/TW	15.0 ± 0.1	16.4 ± 0.1	12.9 ± 0.2
AR/TW	14.0 ± 0.1	38.6 ± 0.2	23.6 ± 0.1
50%EtOH extracts			
SBT/TW	15.5 ± 0.1	46.2 ± 0.1	36.2 ± 0.2
SBT/LV	22.4 ± 0.1	22.6 ± 0.1	0
BC/TW	15.1 ± 0.1	36.1 ± 0.1	33.9 ± 0.2
AR/TW	17.8 ± 0.1	48.6 ± 0.1	74.0 ± 0.3

**Table 5:** Tentative identification of the chemical compounds found in SBT/TW, AR/TW and BC/TW extracts by UHPLC-MS/MS under negative ionization.

No.	tR Diapazone (min), All Spectra	Tentative Compound	*m*/*z*	Error (ppm)	MS/MS
1	0.96	Gluconic acid (2R,3S,4R,5R)-2,3,4,5,6-pentahydroxyhexanoic acid	195.0509	−0.81	177.04; 129.02
2	1.02	Disaccharide	341.1086	−0.93	202.07; 179.05; 89.02; 59.01
3	1.02	Quinic acid ((1R,3S,4S,5S)-3,4-bis[[(E)-3-(3,4-dihydroxyphenyl)prop-2-enoyl]oxy]-1,5-dihydroxycyclohexane-1-carboxylic acid)	191.0561	−0.10	111.01; 87.01; 85.03
4	1.11	Malic acid (2-hydroxybutanedioic acid)	133.0142	−0.45	115.00; 71.01
5	1.53	Citric acid (2-hydroxypropane-1,2,3-tricarboxylic acid)	191.0195	−1.36	111.01
6	2.61	Gallic acid (3,4,5-trihydroxybenzoic acid)	169.0142	−0.53	125.02
7	4.87	Protocatechuic acid 3-glucoside (4-hydroxy-3-[(2S,3R,4S,5S,6R)-3,4,5-trihydroxy-6-(hydroxymethyl)oxan-2-yl]oxybenzoic acid)	315.072	−0.44	631.15; 315.07; 153.02; 152.01; 109.03, 108.02
8	4.99	Protocatechuic acid (3,4-dihydroxybenzoic acid)	153.0193	0.12	153.02; 135.01; 109.03
9	5.02	L-DOPA 3′-glucoside (2-amino-3-[4-hydroxy-3-[3,4,5-trihydroxy-6-(hydroxymethyl)oxan-2-yl]oxyphenyl]propanoic acid)	358.1141	−0.77	312.11; 177.05; 161.05; 150.06; 119.04; 113.03; 101.02; 89.02
10	5.26	Galloyl glucose ([(2S,3R,4S,5S,6R)-3,4,5-trihydroxy-6-(hydroxymethyl)oxan-2-yl] 3,4,5-trihydroxybenzoate)	331.0671	−0.44	313.06; 169.01 168.01; 125.02
11	6.05	Vanillic acid glucoside (3-methoxy-4-[3,4,5-trihydroxy-6-(hydroxymethyl)oxan-2-yl]oxybenzoic acid)	329.0878	0.06	659.18; 167.03
12	6.63	Chlorogenic acid ((1S,3R,4R,5R)-3-[(E)-3-(3,4-dihydroxyphenyl)prop-2-enoyl]oxy-1,4,5-trihydroxycyclohexane-1-carboxylic acid)	353.0876	−0.64	191.05; 179.03
13	6.86	Salicylic acid (2-hydroxybenzoic acid)	137.0244	0.18	93.03
14	7.83	3-Methoxy-4-hydroxyphenylglycol glucuronide ((2S,3S,4S,5R,6R)-3,4,5-trihydroxy-6-[2-hydroxy-2-(4-hydroxy-3-methoxyphenyl)ethoxy]oxane-2-carboxylic acid)	359.0981	−0.63	197.05; 182.02; 153.06; 138.03
15	8.53	Epigallocatechin ((2R,3R)-2-(3,4,5-trihydroxyphenyl)-3,4-dihydro-2H-chromene-3,5,7-triol)	305.0666	−0.30	261.08; 219.07; 219.07; 179.03; 167.04; 137.02; 125.02
16	8.63	Catechin ((2R,3S)-2-(3,4-dihydroxyphenyl)-3,4-dihydro-2H-chromene-3,5,7-triol)	289.0718	0.06	245.08; 205.05; 201.07; 179.04; 125.02; 123.05; 109.03
17	10.02	Neochlorogenic acid ((1R,3R,4S,5R)-3-[(E)-3-(3,4-dihydroxyphenyl)prop-2-enoyl]oxy-1,4,5-trihydroxycyclohexane-1-carboxylic acid)	353.0876	−0.64	191.05
18	10.24	Procyanidin B-type dimer ((2R,3S)-2-(3,4-dihydroxyphenyl)-8-[(2R,3R,4R)-2-(3,4-dihydroxyphenyl)-3,5,7-trihydroxy-3,4-dihydro-2H-chromen-4-yl]-3,4-dihydro-2H-chromene-3,5,7-triol)	577.1348	−0.56	407.07; 289.07; 245.08; 202.08; 161.02; 125.02
19	10.31	Caffeic acid ((E)-3-(3,4-dihydroxyphenyl)prop-2-enoic acid)	179.0349	−0.38	201.07; 135.05
20	10.82	Caffeoylquinic acid ((1S,3R,4R,5R)-3-[3-(3,4-dihydroxyphenyl)prop-2-enoyloxy]-1,5-dihydroxy-4-[3-(4-hydroxy-3-methoxyphenyl)prop-2-enoyloxy]cyclohexane-1-carboxylic acid)	353.0876	−0.64	191.05
21	12.15	Procyanidin B-type trimer ((2R,3R)-2-(3,4-dihydroxyphenyl)-8-[(2R,3R)-2-(3,4-dihydroxyphenyl)-3,5,7-trihydroxy-3,4-dihydro-2H-chromen-4-yl]-4-[(2R,3S)-2-(3,4-dihydroxyphenyl)-3,5,7-trihydroxy-3,4-dihydro-2H-chromen-8-yl]-3,4-dihydro-2H-chromene-3,5,7-triol)	865.1978	−0.84	695.15; 577.14; 451.11; 407.08; 289.07
22	12.23	Epicatechin ((2R,3R)-2-(3,4-dihydroxyphenyl)-3,4-dihydro-2H-chromene-3,5,7-triol)	289.0716	−0.57	245.08; 205.05; 201.07; 179.04; 125.02; 123.05; 109.03
23	12.41	Procyanidin B-type tetramer ((2R,3S,4R)-2-(3,4-dihydroxyphenyl)-4-[(2R,3S)-2-(3,4-dihydroxyphenyl)-3,5,7-trihydroxy-3,4-dihydro-2H-1-benzopyran-8-yl]-8-[(2R,3S,4S)-2-(3,4-dihydroxyphenyl)-8-[(2R,3S,4S)-2-(3,4-dihydroxyphenyl)-3,5,7-trihydroxy-3,4-dihydro-2H-1-benzopyran-4-yl]-3,5,7-trihydroxy-3,4-dihydro-2H-1-benzopyran-4-yl]-3,4-dihydro-2H-1-benzopyran-3,5,7-triol)	1153.2623	0.35	577.13; 525.08; 449.09; 407.08; 287.06; 243.03; 161.02
24	13.70	p-coumaric acid ((E)-3-(4-hydroxyphenyl)prop-2-enoic acid)	163.0401	0.09	119.05
25	18.95	Rutin (2-(3,4-dihydroxyphenyl)-5,7-dihydroxy-3-[(2S,3R,4S,5S,6R)-3,4,5-trihydroxy-6-[[(2R,3R,4R,5R,6S)-3,4,5-trihydroxy-6-methyloxan-2-yl]oxymethyl]oxan-2-yl]oxychromen-4-one)	609.1457	−0.69	163.00; 151.00; 148.02; 135.00
26	19.87	Myricetin (3,5,7-trihydroxy-2-(3,4,5-trihydroxyphenyl)chromen-4-one)	317.03	−0.73	151.00; 137.02; 109.03
27	21.80	Quercetin (2-(3,4-dihydroxyphenyl)-3,5,7-trihydroxychromen-4-one)	301.0351	−1.05	273.04; 201.07; 151.00; 121.03
28	23.28	Kaempferol (3,5,7-trihydroxy-2-(4-hydroxyphenyl)chromen-4-one)	285.04	−0.19	159.05; 151.00; 143.05; 117.03

**Table 6:** Hydrophilic extracts and individual compounds influence the oxidative stability of creams.

Sample	IP *, h	PF **	IP *, h	PF **
	Lipid Content in LBS–19%		Lipid Content in LBS–35%	
Cream base without antioxidants	11.0	1.0	1.9	1.0
SBT/TW/AR/TW/BC/TW water extracts				
Cream + 0.5% extract on LBS	28.2/31.6/26.4	2.6/2.9/2.4	3.6/4.3/3.2	1.9/2.3/1.7
Cream + 1% extract on LBS	32.5/34.2/28.2	3.0/3.1/2.6	3.8/4.8/3.6	2.0/2.5/2.1
Cream + 2% extract on LBS	37.3/39.6/36.7	3.4/3.6/3.3	4.2/4.6/3.8	2.2/2.4/2.0
SBT/TW/AR/TW/BC/TW 50% EtOH extract				
Cream + 0.5% extract on LBS	29.5/31.6/27.4	2.8/3.0/2.6	4.1/4.4/3.8	2.2/2.3/2.0
Cream + 1% extract on LBS	34.1/36.6/32.2	3.1/3.3/2.9	3.4/3.6/3.2	1.8/1.9/1.7
Cream + 2% extract on LBS	36.8/43.2/32.4	3.4/3.9/3.0	4.4/4.7/4.0	2.3/2.5/1.6
SBT/AR/BC purified PACs				
Cream + 0.5% PACs on LBS	n.a ***	n.a	3.5/3.6/3.4	1.8/1.9/1.8
SBT/TW/AR/TW/BC/TW biomass (from Table 1)				
Cream + 2% extract on LBS		1.5/1.7/1.3		1.1/1.2/1.1
Gallic acid (GA)				
Cream + 0.5% GA on LBS	n.a	n.a	2.0	1.1
Cream + 1% GA on LBS	n.a	n.a	3.9	2.1
Ascorbic acid (AA)				
Cream + 0.5% AA on LBS	n.a	n.a	1.9	1.0
Cream + 1% AA on LBS	n.a	n.a	2.1	1.1
TBHQ				
Cream + 1% TBHQ on LBS	-	-	-	1.3 [67]
Cream + 2% TBHQ on LBS	-	-	-	1.8 [67]
Cream + 3% TBHQ on LBS	-	-	-	2.4 [67]

* IP—the induction period (time, h) of cream oxidation when there is a sample in the cream; ** PF—the protection factor. *** n.a.—not available. At α = 0.05, the confidence interval for every result was less than 0.2.

**Table 7:** Antimicrobial activity of PACs and hydrophilic extracts in comparison with synthetic antibiotics.

Samples	*P. aeruginosa*	*S. aureus*	*B. cereus*	*S. pyogenes*	*C. acnes*
	MIC */MBC **, mg·mL^−1^				
SBT/TW water extract	0.39/3.13	0.39/0.78	0.78/>50	0.20/0.20	0.78/0.78
AR/TW water extract	2.5/>5.0	0.039/0.31	0.16/0.16	0.20/0.20	1.56/1.56
BC/TW water extract	1.25/1.25	0.63/0.63	1.25/>5.0	n.d.	n.d.
SBT/TW 50% EtOH extract	0.39/0.78	0.20/0.39	0.39/50	0.20/0.20	0.39/0.39
AR/TW 50% EtOH extract	0.63/2.5	0.31/0.63	0.16/>5.0	0.05/0.05	1.56/1.56
BC 50% EtOH extract	0.63/0.63	2.5/2.5	1.25/>5	n.d. ***	n.d.
SBT/TW purified PACs	0.08/0.16	0.08/0.16	0.63/1.25	0.10/0.10	0.39/0.39
AR/TW purified PACs	0.16/0.63	0.08/0.08	0.04/0.08	0.08/0.08	2.50/2.50
BC/TW purified PACs				0.08/0.08	2.50/5.0
Amoxicillin	-	0.13/0.13	0.008/0.016 ****	-	0.0001 ******
Bactroban	0.16/0.18 *****	0.20/0.20 *****	-	0.20/0.40 *****	-
Gentamicin	0.0003/0.004	0.0003/0.004	-	0.40/0.40 *****	0.004 ******
Chlor-amphenicol	0.40/0.40 *****	0.40/0.40 *****	-	0.40/0.40 *****	-

The confidence interval for all the results did not exceed 0.05 mg·mL^−1^ at α = 0.05. * MIC—Minimum Inhibitory Concentration; ** MBC—Minimum Bactericidal Concentration; *** n.d.—not detected; **** [68]; ***** [69]; ****** [70].

## Data Availability

The raw data supporting the results of this article will be available by the authors on request.

## Supplementary Materials

The following supporting information can be downloaded at: https://www.mdpi.com/article/10.3390/ijms27020701/s1.

## Abbreviations

The following abbreviations are used in this manuscript:

SBT	sea buckthorn
AR	Aronia
BC	Blackcurrant
TW	Twigs
LV	Leaves
LBS	lipid-bases system
PACs	Proanthocyanidins
GA	gallic acid
AA	ascorbic acid
DB	dry biomass
DE	dry extract
