# Supplementary material for: Sea Buckthorn, Aronia, and Black Currant Pruning Waste Biomass as a Source of Multifunctional Skin-Protecting Cosmetic and Pharmaceutical Cream Ingredients
Source: Int J Mol Sci. 2026 Jan 9;27(2):701. doi: 10.3390/ijms27020701 (PMC12840945; doi:10.3390/ijms27020701)
Supplement: Supplementary file 1 [file ijms-27-00701-s001.zip › ijms-4049288-supplementary.pdf]

Supplementary Materials

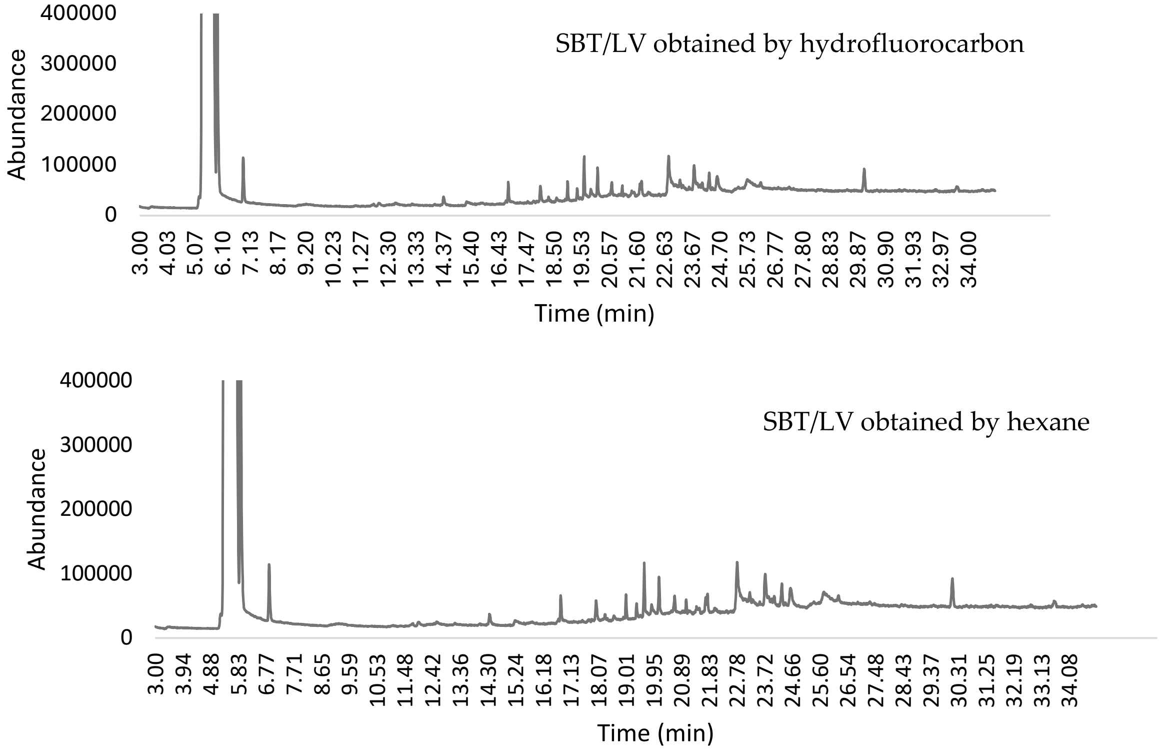

Figure S1. GC/MS/FID spectra for hydrofluorocarbon and hexane extract of SBT/LV

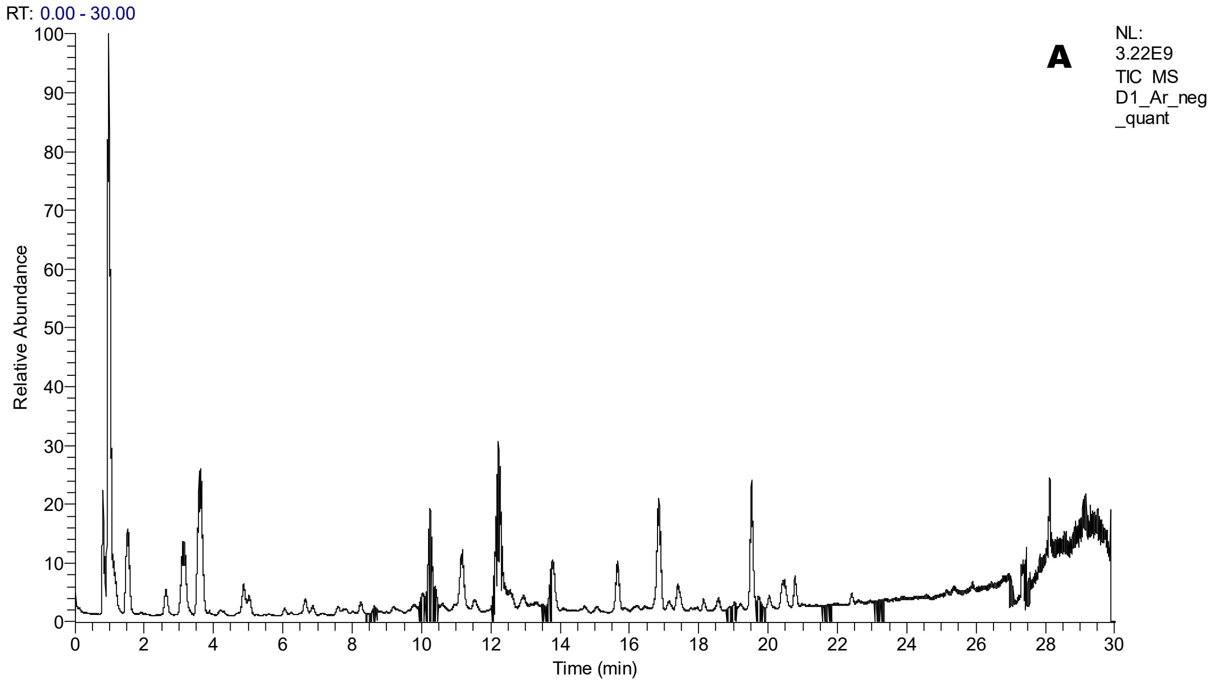

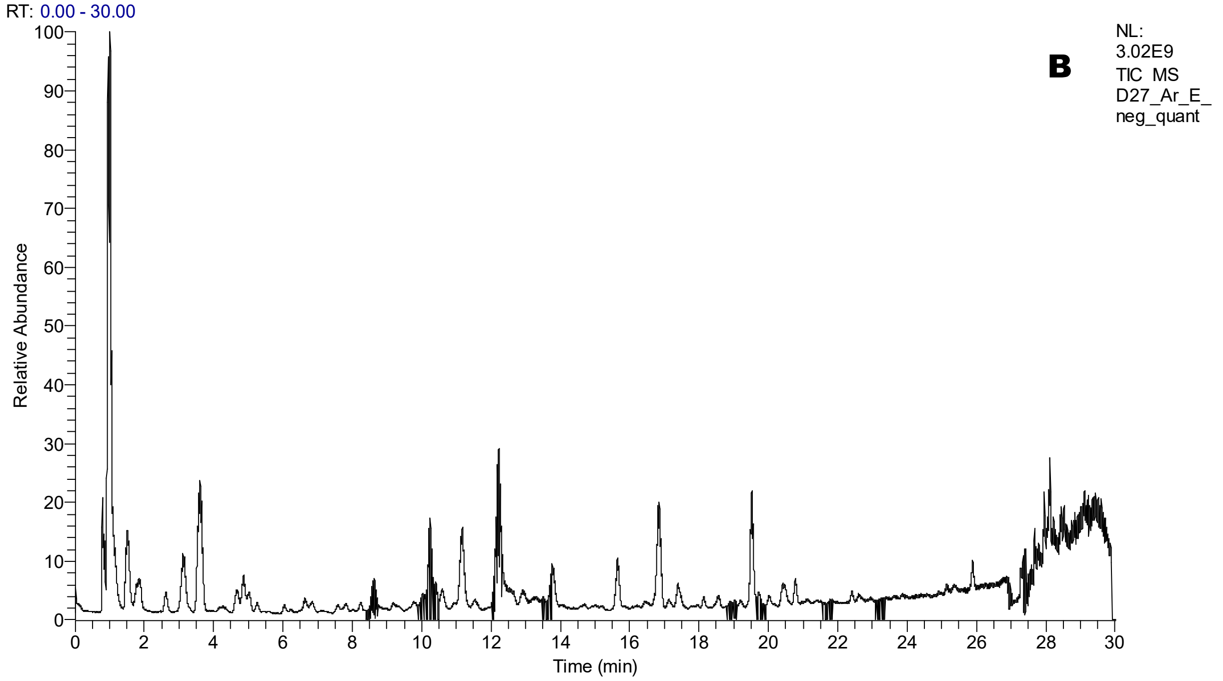

RT: 0.00 - 30.00
B
NL:
3.02E9
TIC MS
D27_Ar_E_
neg_quant
Relative Abundance
Time (min)
0
10
20
30
40
50
60
70
80
90
100
0
2
4
6
8
10
12
14
16
18
20
22
24
26
28
30

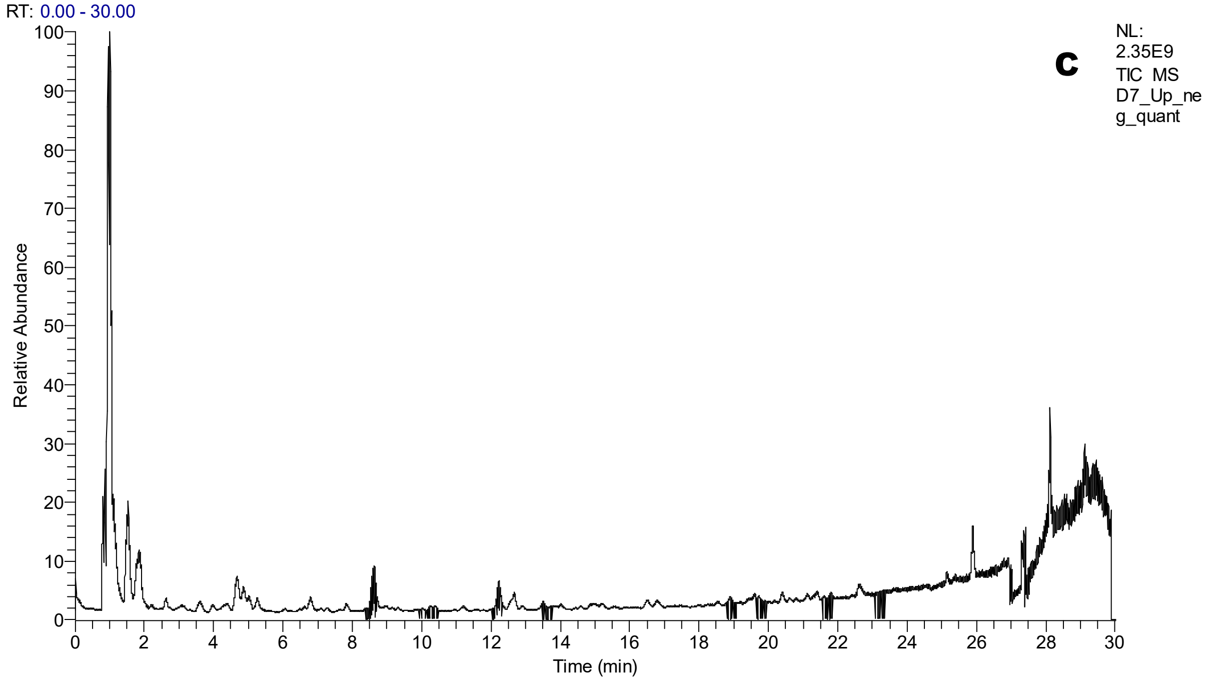
RT: 0.00 - 30.00
NL:
2.35E9
TIC MS
D7_Up_ne
g_quant
C
Relative Abundance
Time (min)

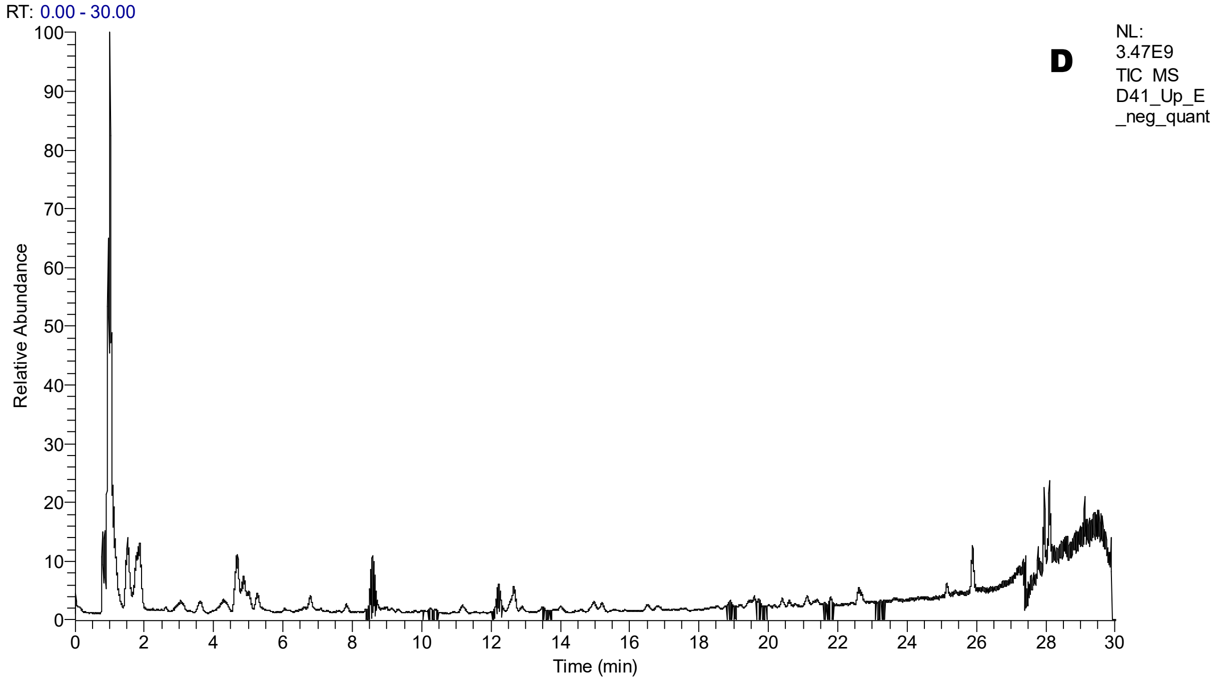
RT: 0.00 - 30.00
NL:
3.47E9
TIC MS
D41_Up_E
_neg_quant
D
Relative Abundance
Time (min)

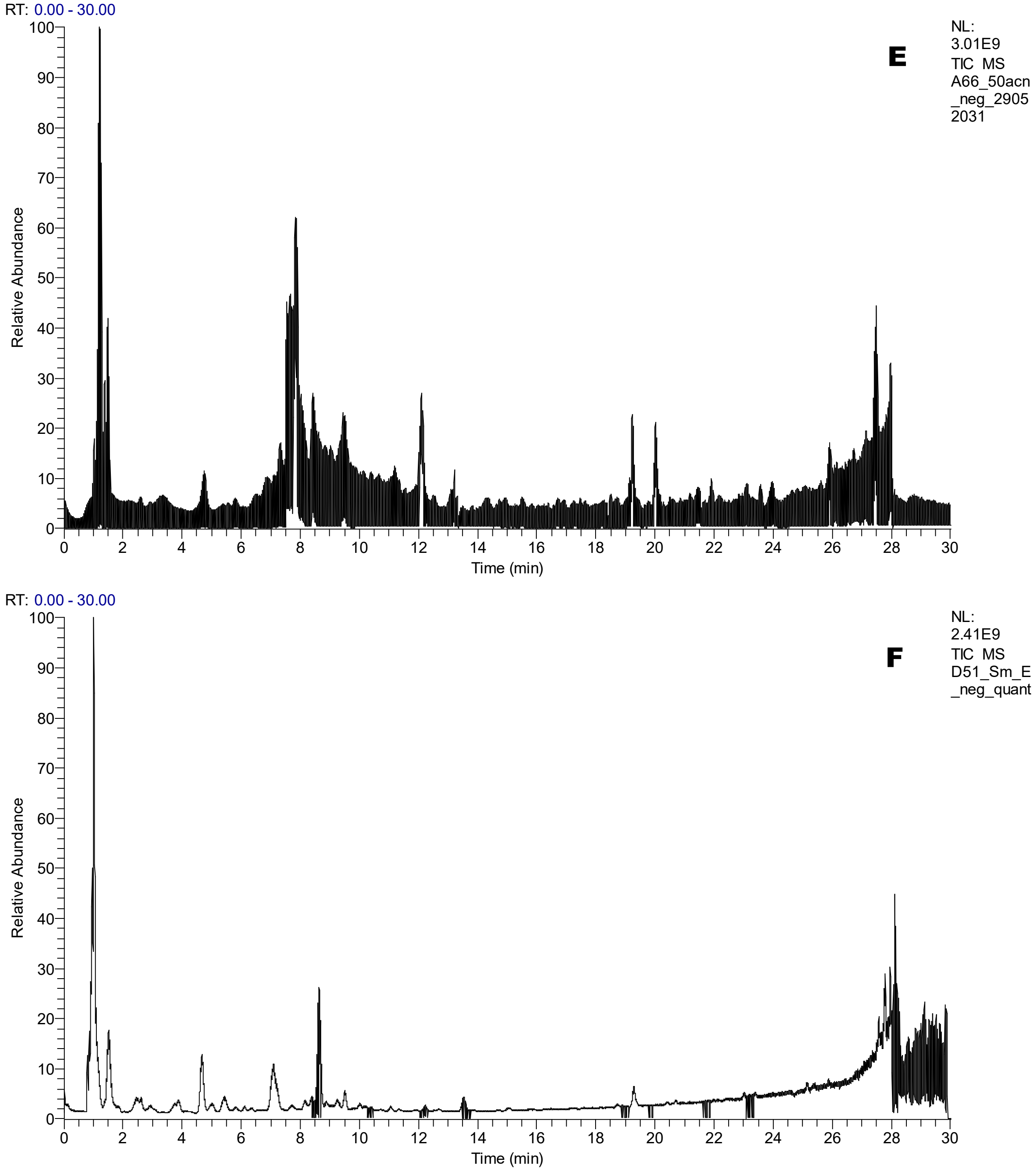

Figure S2. LC-Orbitrap-MS/MS chromatograms of BC/TW water (**A**) and 50% EtOH (**B**) extracts; AR/TW water (**C**) and 50% EtOH extracts (**D**); SBT/TW water (**E**) and 50% EtOH extracts (**F**) recorded in total ion mode

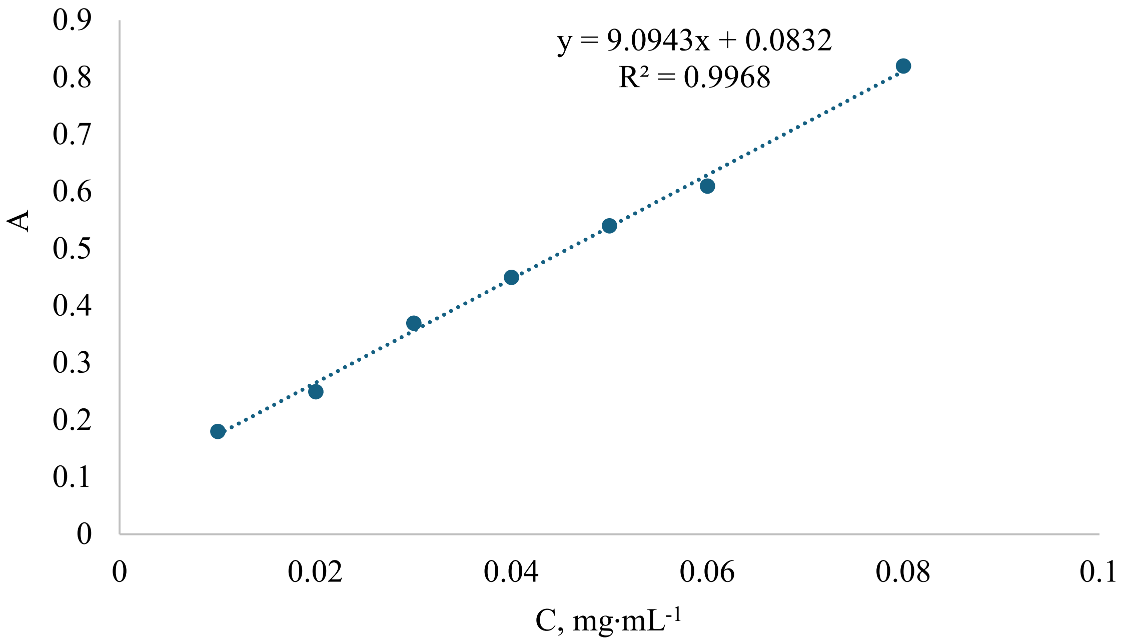

Figure S3. Standard calibration curve (gallic acid) for determination of total polyphenols content in the hydrophilic extract.

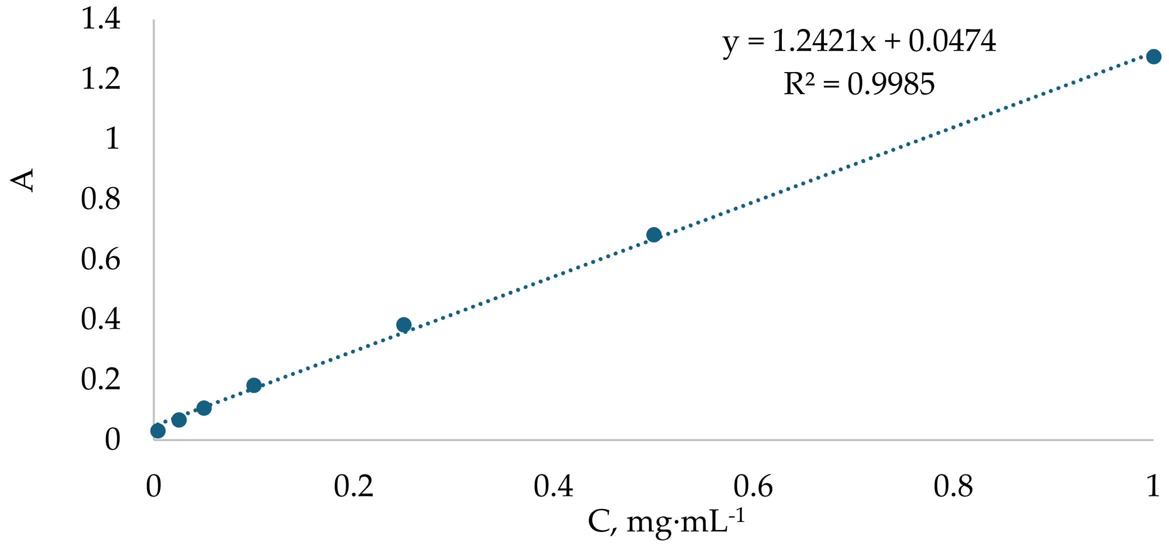

Figure S4. Standard calibration curve (procyanidin dimer B2 standard) for determination proanthocyanidins amount in the hydrophilic extract.

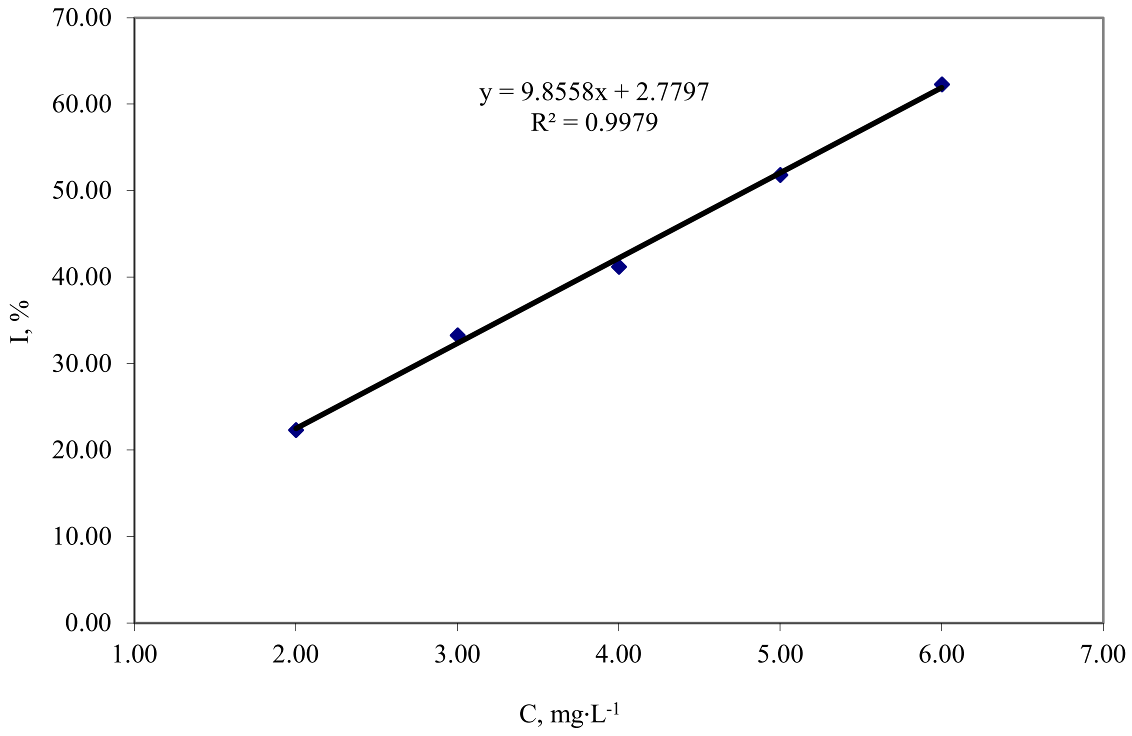

Figure S5. Inhibition of DPPH radical depending on Trolox concentration.

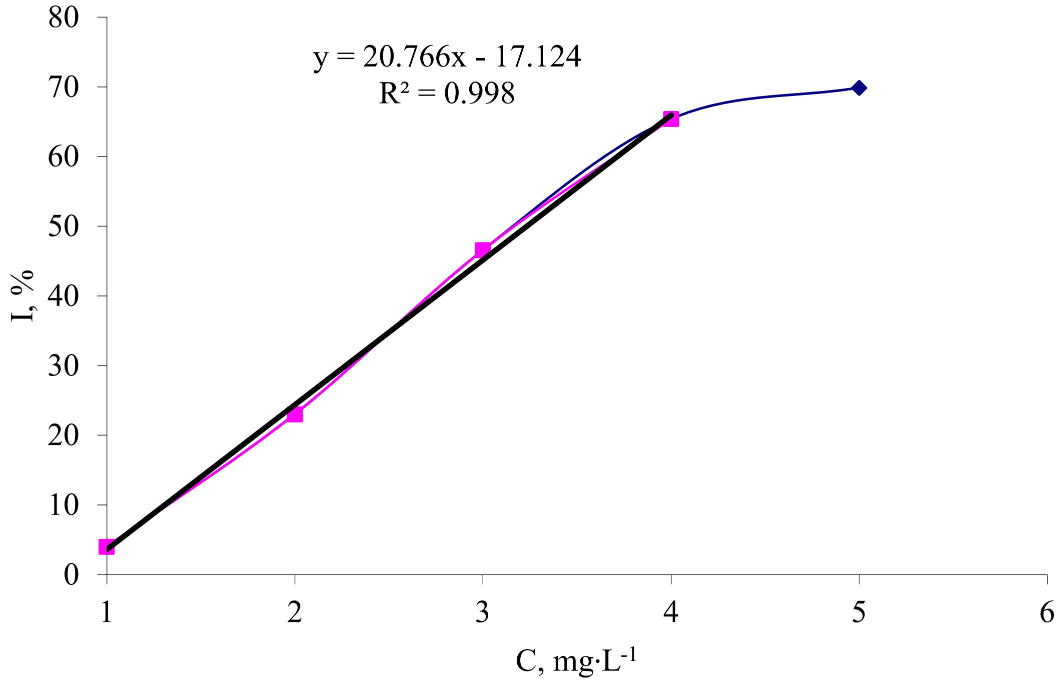

Figure S6. Inhibition (I) of $ABTS^+$ radical depending on Trolox concentration.
